# Recent Advances in Catalysis Involving Bidentate N-Heterocyclic Carbene Ligands

**DOI:** 10.3390/molecules27010095

**Published:** 2021-12-24

**Authors:** Abdollah Neshat, Piero Mastrorilli, Ali Mousavizadeh Mobarakeh

**Affiliations:** 1 Department of Chemistry, Institute for Advanced Studies in Basic Sciences (IASBS), Zanjan 45137-66731, Iran; alimousavi@iasbs.ac.ir; 2 Dipartimento di Ingegneria Civile, Ambientale, del Territorio, Edile e di Chimica, Politecnico di Bari, Via Orabona, I-70125 Bari, Italy; p.mastrorilli@poliba.it

**Keywords:** N-heterocyclic carbenes, catalysis, chiral, coupling reactions, transfer hydrogenation, polymerization

## Abstract

Since the discovery of persistent carbenes by the isolation of 1,3-di-l-adamantylimidazol-2-ylidene by Arduengo and coworkers, we witnessed a fast growth in the design and applications of this class of ligands and their metal complexes. Modular synthesis and ease of electronic and steric adjustability made this class of sigma donors highly popular among chemists. While the nature of the metal-carbon bond in transition metal complexes bearing N-heterocyclic carbenes (NHCs) is predominantly considered to be neutral sigma or dative bonds, the strength of the bond is highly dependent on the energy match between the highest occupied molecular orbital (HOMO) of the NHC ligand and that of the metal ion. Because of their versatility, the coordination chemistry of NHC ligands with was explored with almost all transition metal ions. Other than the transition metals, NHCs are also capable of establishing a chemical bond with the main group elements. The advances in the catalytic applications of the NHC ligands linked with a second tether are discussed. For clarity, more frequently targeted catalytic reactions are considered first. Carbon–carbon coupling reactions, transfer hydrogenation of alkenes and carbonyl compounds, ketone hydrosilylation, and chiral catalysis are among highly popular reactions. Areas where the efficacy of the NHC based catalytic systems were explored to a lesser extent include CO_2_ reduction, C-H borylation, alkyl amination, and hydroamination reactions. Furthermore, the synthesis and applications of transition metal complexes are covered.

## 1. Introduction

The current concept of coordination bond in the metal complexes defies the earlier definition of the covalent bond in molecular structures. While the latter definition, based on the covalent bond theory, relies on electron sharing between bond partners, the donation of a lone pair of electrons to a lone pair electron acceptor occurs in coordination compounds. This definition also includes the formation of double and triple bonds in coordination compounds. While the majority of nonmetals, including small monoatomic species such as fluoride ions, capable of donating electron pairs, are considered ligands, one of the most fascinating adventures in modern coordination chemistry occurred following the isolation of Arduengo’s stable and free NHC in 1991 by the deprotonation of an imidazolium salt [1]. Following advances made in the preparation of stable NHCs, donor functionalized NHCs, such as those developed by Hermann in 1996, paved the way for a fascinating chemistry involving NHCs, well suited for practical applications such as catalytic reactions [2]. Among donor functionalized carbene ligands, those that contain acidic functional groups such as amines or alcohols acquired more popularity because of their readily accessible chemistry [3].

In many aspects, the NHCs bear resemblance to phosphine ligands. They offer a strong σ-donation to metal ions, their electronic and steric properties are tunable by varying the ligand substituents, and both are weak π-acceptors. However, NHC ligands are relatively more reactive ligands because they can get involved in ligand modification at the metal coordination sphere. Some fundamental reactions involving NHCs include insertion and reductive elimination [4,5,6].

**Figure 1:**
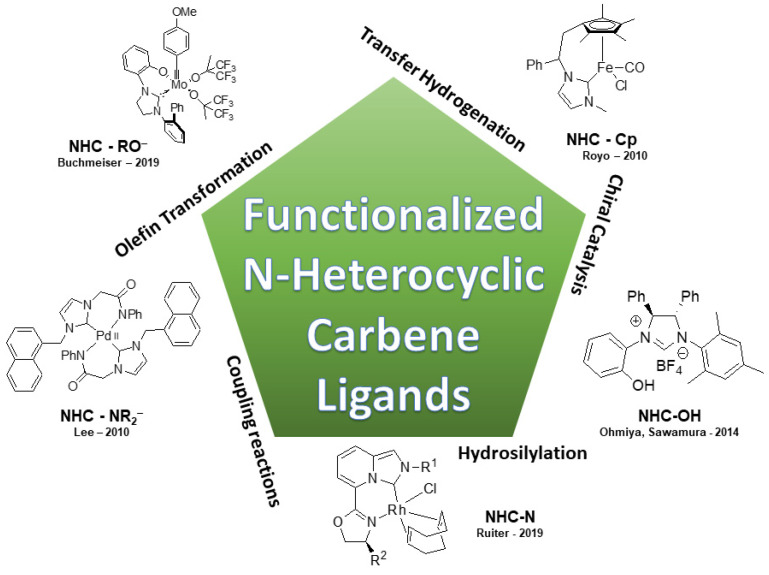
Selected examples of tethering bidentate NHCs ligands bearing neutral or anionic moieties.

There are several review articles reported in the past decade, which are dedicated to NHC ligands bearing one additional donor group [7,8,9,10]. Included in these review articles are bidentate NHC ligands with phosphorous tether [11,12,13,14,15,16,17,18,19], nitrogen [20,21,22,23,24,25,26,27,28,29,30,31,32,33,34,35,36,37], and oxygen tethers [38,39,40,41,42,43,44,45,46,47,48,49,50,51,52,53,54,55,56,57,58,59,60,61,62,63,64,65,66,67,68,69,70,71,72,73,74,75,76,77,78,79,80,81,82,83,84,85,86,87,88,89,90,91,92,93,94,95,96,97,98,99,100,101,102,103,104,105,106]. A review article dedicated to sulfur functionalized NHC ligands was reported in 2014 [107]. These interesting ligand systems were utilized in a wide range of applications. Some of these include biological and catalysis sciences. This prompted us to write a short review article addressing catalytic applications of bidentate ligands containing the NHC ligand as the main electron donor site. In particular, synthesis and the way these complexes are applied in various catalytic reactions will be the main focus of this review article. The synthesis, metal complexation and applications of bidentate NHC ligands bearing neutral or anionic side chains will be discussed for the time span of about 10 years from 2010. The side chains of the NHCs, also known as “tether” atoms/groups or substituents can be either neutral or anionic donor atoms, which confer some novel properties to the NHC ligands by turning monodentate NHCs into chelating (and therefore more stable) ligands. In addition, by placing a chiral center in the side chain of a bidentate NHC ligand, one can explore its potential in catalytic asymmetric reactions. Figure 1 displays five types of bidentate NHCs ligands with a range of catalytic applications. 

## 2. Synthesis and Applications of Bidentate Ligands Bearing N-Heterocyclic Carbenes

### 2.1. Hydrogenation Reactions of Carbonyl Compounds, Alkene, and Alkynes

Hydrogenation of organic functional groups with multiple bonds is considered one of the most fundamental reactions in chemistry. Traditionally, this process was carried out under high pressure H_2_ gas with Lewis acidic or Basic catalysts at high temperatures. A more convenient method of hydrogenation is carried out via transfer hydrogenation routes. Transfer hydrogenation (TH), that is the addition of hydrogen to a substrate not using hydrogen gas as reagent, is a convenient method to access various hydrogenated compounds. There are many benefits regarding the use of a non-H_2_ source for hydrogenation reactions compared to the more conventional methods of using pressured hydrogen gas which is hazardous and requires elaborate experimental setups. Most hydrogen donors employed for TH reactions, such as 2-propanol or formic acid, are commercially available and inexpensive [108].

Although, details regarding TH reactions are great and excellent review articles were published in recent years, the late transition metal catalyzed TH reactions set a milestone in the catalytic TH reactions. The earlier examples of literature reports regarding transition metal catalysts benefited from phosphine-based ligands [109]. First examples of NHC based transition metal complexes were introduced three decades later in 2001 by the pioneering works of Nolan et al., who introduced Iridium(I) complexes with monodentate NHC ligands [110]. Because of higher stability and the possibility of catalyst tunability both in reactivity and selectivity, bidentate NHC ligands were also extensively investigated in these reactions. Prominent examples in this regard involve the work of Crabtree and Royo [111,112]. From the beginning of last decade and within 2010–2021, we saw a continued growth in the quality and quantity of research papers concerning TH of organic substrates. Examples of catalytic systems developed for the hydrogenation reactions of alkene, alkyne and carbonyl compounds using bidentate nitrogen-containing heterocyclic carbene (NHC) linked with another tether ligand are as follow. Because of interesting synthetic protocols in bidentate NHC ligand design and metalation procedures, in some cases these procedures are also described along with critical review of each catalytic reaction.

**Scheme 1:**
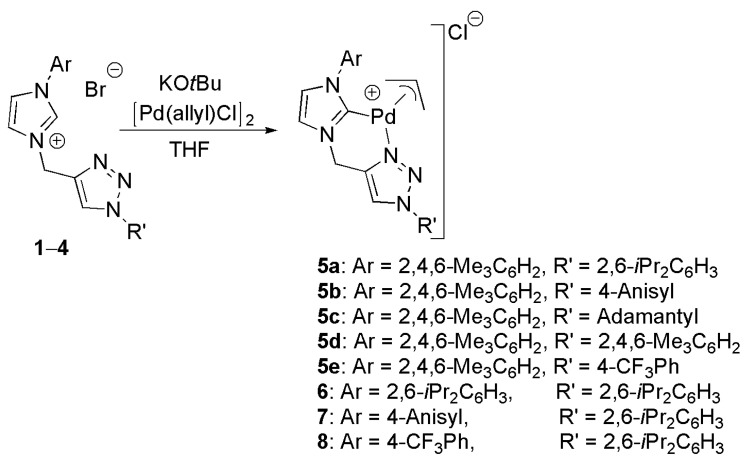
“Direct method” of metalation of a bidentate NHC ligands with tethered triazolyl to support palladium (II) ions [113].

Elsevier et al. introduced a general procedure for the synthesis of a novel bidentate NHC-triazolyl donor in 2010. The imidazolium salts **1**–**4** (Scheme 1) are prepared in two steps. Firstly, an *N*-arylimidazole is reacted with propargyl bromide in acetonitrile to form alkynyl-imidazolium. Then, “click reaction” of the resulting salt, in the presence of catalytic amount of copper sulphate, yielded the desired NHC with 1,2,3-triazolyl tether [113].

**Scheme 2:**
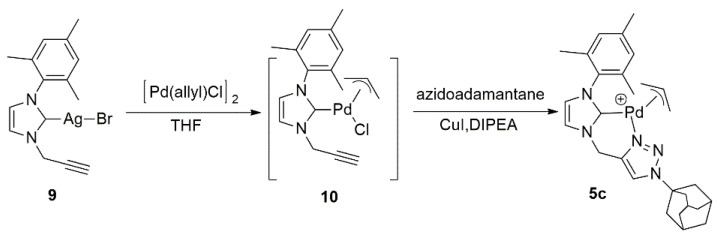
“Modular Approach” to synthesis palladium complexes of a bidentate NHC ligands with tethered triazolyl group [113]. DIPEA = *N*,*N*-diisopropylethylamine.

A direct approach to the metalation of these ligands involves typical deprotonation of imidazolium salt with potassium tert-butoxide and subsequent addition of the metal salt of choice, palladium allyl chloride in Scheme 2. The direct metal complexation of bidentate ligands was shown to work for a variety of substituents on *N*-aryls and R’ substituents on triazole ring leading to Pd complexes **5**–**8**. 

Scheme 2 shows that, in some cases, the synthesis of NHC-palladium complexes could be accomplished directly and synthesis of silver complex intermediate and subsequent transmetalation with palladium could be disregarded. Therefore, reaction steps will be reduced. 

The synthesis of the palladium complex **5c** could be achieved by the strategy depicted in Scheme 2: transmetalation of the silver complex **9** with [Pd(allyl)Cl]_2_ afforded complex **10** which, on turn, transformed into **5c** by a “click” reaction with azidoadamantane [113]. This strategy permitted to reduce the number of reaction steps required for the synthesis of a library of palladium complexes.

**Scheme 3:**
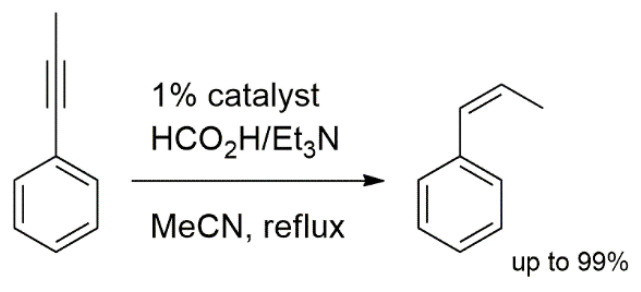
Transfer hydrogenation of alkynes catalyzed by Pd(NHC)allyl complexes [113].

These novel palladium complexes were utilized in the catalytic semi-TH of selected alkynes to Z-alkenes. Despite having a slow rate of reaction compared to that of previously reported palladium catalysts with mono-dentate NHC ligands, the substrates **1**–**4** showed high selectivity of up to 99%. The model reaction for the alkyne semihydrogenation reported is shown in Scheme 3. 

**Scheme 4:**
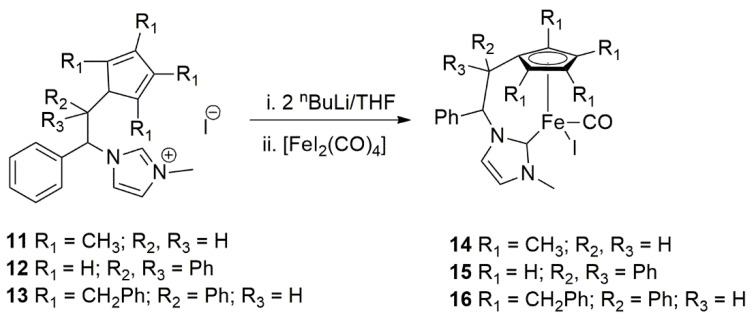
In situ generation of piano-stool iron(II) complexes featuring bidentate NHC-C_p_ donors [116].

In late transition metal chemistry, literature reports regarding catalytic applications of iron complexes with NHC ligands are rare. The first example including an NHC ligand in homogeneous iron catalysis dates back to the year 2000, when Grubbs et al. described the use of homogeneous iron complexes bearing bidentate NHC ligands in atom transfer radical polymerization of styrene [114]. The use of iron organometallic complexes in the catalytic homogeneous hydrogenation of ketones using H_2_ gas was reported by Casey et al. and several other groups [115]. Apparently the first report on the catalytic TH of ketones using well-defined iron(II)-NHC complexes published by Royo at al. in 2010, which utilized both Cp and NHC ligands to support iron center in catalytic TH reaction [116]. To stabilize transition metal ions such as iron(II) for catalytic applications, cyclopentadienyl tethered NHC ligands were obtained by simultaneous deprotonation of diene ring and imidazolium by reacting proligands **11**–**13** with two equivalents of *n*-butyllithium (Scheme 4). Addition of one equivalent of [FeI_2_(CO)_4_] afforded green colored air stable piano-stool iron(II) complexes **14**–**16** (Scheme 4) [116].

**Scheme 5:**
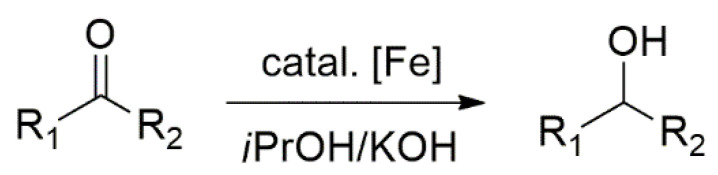
Catalytic transfer hydrogenation catalyzed by complexes **14** and **16** [116].

Single-crystal X-ray diffraction analysis showed that complexes **15** and **16** possess a distorted three-legged piano stool geometry with the cyclopentadienyl-NHC ligand chelating the iron(II) center. These complexes were shown to be excellent catalysts in TH and hydrosilylation of ketones [116]. The catalytic TH of acetophenone, cyclohexanone and benzophenone, Scheme 5, were carried out in 2-propanol, which serves as a hydrogen source. The complexes **14**–**16** catalyzed the hydrogenation of these ketones via hydrogen transfer from *i*PrOH/KOH at 80 °C. In all cases, the reported yields were higher than 75%. The advantageous of this catalyst, despite requiring 1.0 mol% of the catalyst, is a shorter reaction time and implementing KOH, which is more economical than commonly used *t*BuOK. A comparison of the catalytic activity of these complexes also showed that **14**–**16** are close in catalytic activity and that Cp substituents does not seem to affect the catalytic reaction outcome.

**Scheme 6:**
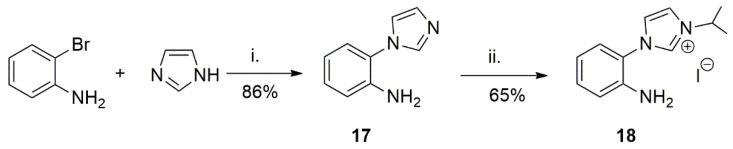
Synthesis of proligand **18**. [117]. Reagents and conditions: (i) Cu_2_O (5 mol%), 8-hydroxyquinoline (20 mol%), Cs_2_CO_3_, MeCN, 100 °C (ii) *i*Prl, MeCN, 90 °C.

A primary amine tethered NHC ligand was utilized as a supporting ligand to a half sandwich *p*-cymene Ru(II) complex to provide a catalyst precursor for catalytic TH of acetophenone [117]. The procedure to synthesize the ligand involves dehydrohalogenation of imidazole and a primary amine with the use of strong base, Scheme 6. In the second step, alkylation of second nitrogen on **17** provides the desired functionalized imidazolium salt **18**. For the metalation of the proligand, Cross et al. achieved the desired product by the reaction of ruthenium arene precursor with a base in the presence of silver oxide as transmetalation reagent. 

**Scheme 7:**
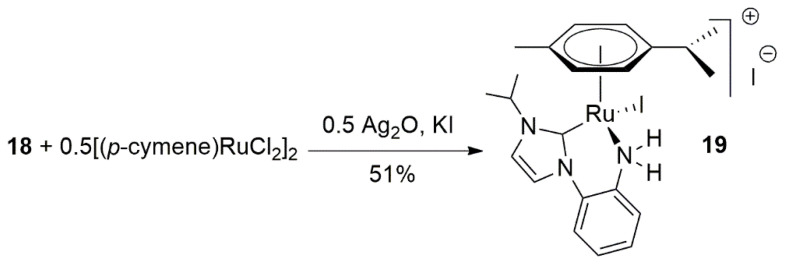
Synthesis of ruthenium NHC-amine complex **19** [117].

The Ru(II) complex **19** in Scheme 7, showed excellent activity (above 90% yield) in the TH of acetophenone over a short reaction time (0.5 h) at refluxing temperature of *i*PrOH. It is notable that Rh and Ir complexes with **18** were also synthesized and their reactivities were tested in catalytic TH reaction. Overall, activity of **19** was higher [117].

**Scheme 8:**
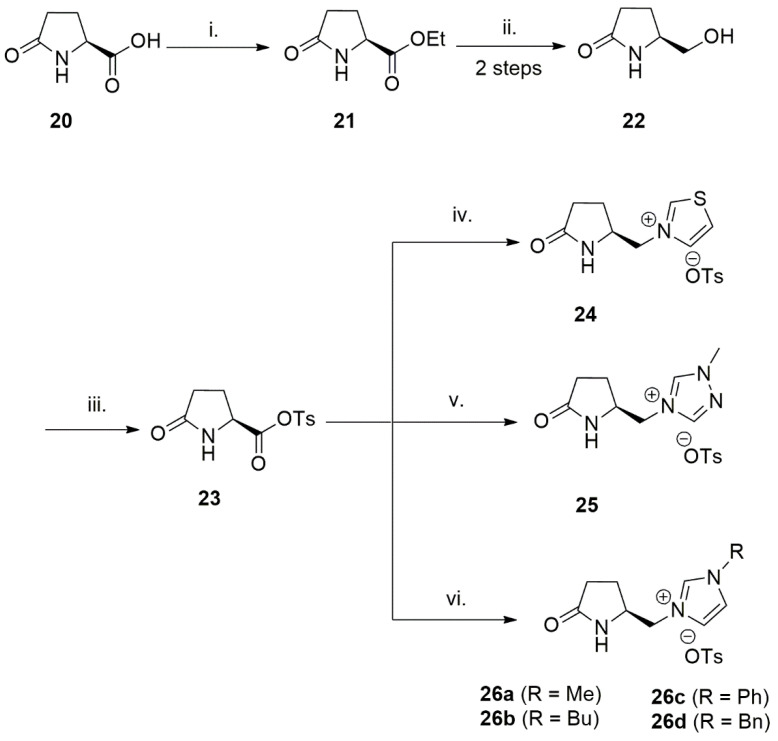
Azolium salts containing a chiral moiety. Reagents and conditions: (i) SOCl_2_, EtOH, 3 h, RT; (ii) NaBH_4_, EtOH, 48 h, RT; (iii) TsCl, Et_3_N, CH_2_Cl_2_, DMAP, 20 h, RT; (iv) thiazole, MW, 130 °C, 2 h; (v) 1-methyl-1,2,4-triazole, MW, 130 °C, 2 h; (vi) 1-substituted-imidazole, MW, 130 °C, 1.5 h. [118].

Synthesis and applications of bidentate NHC ligands with anionic amide group were well established in hydrogenation reactions using a late transition metal [118]. For example, rhodium complexes of these ligands, generated in situ, were utilized in catalytic asymmetric TH reactions of ketones to generate enantiomerically enriched secondary alcohols. The synthetic procedure for the bidentate NHC-amide donors is shown in Scheme 8. Starting out with the esterification of (*S*)-pyroglutamic acid **20**, reduction with NaBH_4_ is then followed to afford alcohol **22**. Tosylated substrate **23** is then converted to the relevant salt by reacting it with thiazole, triazole, or imidazole under microwave irradiation [118]. 

Out of six N-heterocyclic carbene ligands investigated for TH reactions of ketones, surprisingly only **26a** and **26b** with an alkyl substituent on heterocycle nitrogen showed high activity (90% yield in both cases) and enantioselectivities of 80% and 46%, respectively [118]. The main catalyst was generated using [Rh(cod)Cl]_2_ as metal source and the best ratio of the ligand to metal source determined to be 3. Also, potassium hydroxide determined to be the optimum base for the catalytic TH reaction. In this enantioselective catalytic reactions, Vo-Thanh et al. also presented a well-defined catalytic asymmetric TH catalyst with highest enantioselectivity reported in 2011. As expected, with electron rich substrates, such as those containing methoxy substituents, slower reaction rates and higher enantioselectivities were observed.

**Scheme 9:**
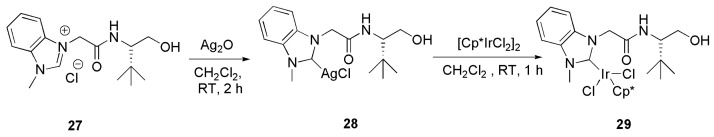
Synthesis of precatalyst **29** [119]. Cp* = Me_5_-Cp.

Base-induced transformation of a monodentate NHC ligand, with a tethered amide, into a chelating NHC-amidate ligand was described by Sakaguchi et al. in 2012 (Scheme 9) [119]. Metalation of the imidazolium salt **27** via standard silver oxide route with Cp*IrCl_2_ yielded the Ir(III) complex **29** which was utilized as precatalyst in catalytic asymmetric TH of acetophenone with moderate yield and enantioselectivity. The reaction required the presence of a base such as potassium hydroxide to proceed. Although XRD analysis of **29** has revealed that NHC ligand is coordinated in a monodentate fashion, the observed base-induced increase in the enantioselectivity of the catalyst was imputed to chelate effect of NHC ligand arising by deprotonation of side chain hydroxyl-amide functional group. 

**Figure 2:**
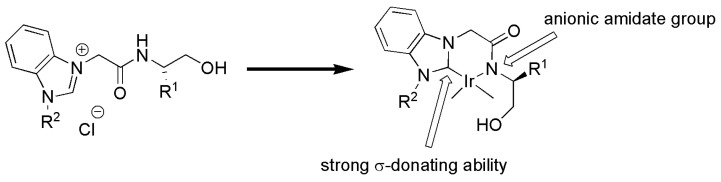
Proposed structure of iridium complex with NHC-amidate ligand [119].

Basically, chelation of NHC ligand through side chain, promoted by a base, locks the chiral side chain in a fixed position, thus allowing catalytic reaction with a preference toward specific enantiomer. The general structure of proposed iridium complex with bidentate NHC-amidate ligand, as a result of deprotonation of **19**, is shown in Figure 2. 

Therefore, under basic conditions and the generation of bidentate form of the NHC ligand, the use of proper substituents on the stereogenic center (R^1^) and (R^2^) on azolium ring, the enantiomeric excess of the reaction was increased up to 60%. 

**Figure 3:**
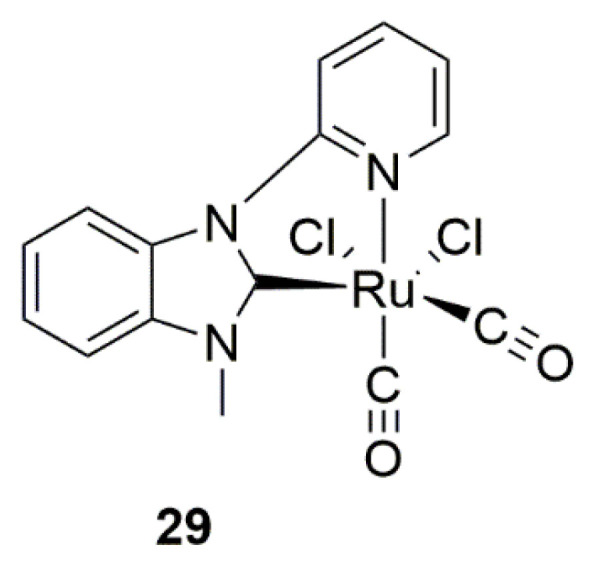
Ru(II) carbonyl complexes investigated in transfer hydrogenation reaction [120].

In the same year, a series of Ru(II) complexes with functionalized NHC ligands were introduced by different research groups. Among these functionalized NHC ligands, phosphine, and also neutral or anionic nitrogen donors were included to provide TH catalysts. For example, Chen et al. reported the synthesis of a series of six coordinated Ru(II) carbonyl complexes bearing an NHC linked with a pyridine tether, Figure 3 [120]. The catalytic activities of these metal complexes in TH of acetophenone under standard reaction conditions (KOH, *i*PrOH as proton donor and reflux) revealed that despite structural similarities of three types of NHC ligands, the catalyst with a better sigma donor NHC ligand, based on imidazole, showed higher catalytic activity (97% yield of alcohol in 2 h) in the hydrogenation reaction. Besides, the presence of a spacer between pyridine tether and nitrogen atom of the NHC ligand decreased the catalytic activity of the catalyst. 

Likewise, the synthesis of a series of novel picolyl NHC ligands and related heteroleptic pentamethylcyclopentadienyl ruthenium Ru(II) complexes was reported in 2011 [121]. These ruthenium complexes catalyzed TH of ketones and imines using *i*PrOH in the presence of KOH. 

**Scheme 10:**
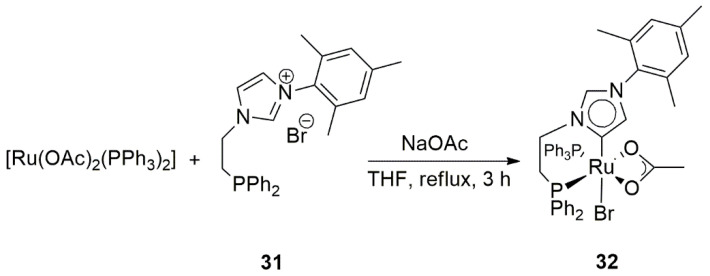
Chelation of bidentate NHC-PR_2_ ligand [122].

The novel octahedral Ru(II) complex **32** bearing “abnormal” or mesoionic bidentate NHC-PPh_2_ ligand (Scheme 10) was developed by Baratta et al. in 2013 [122]. It showed excellent catalytic activity in the TH of ketones with 0.05 mol% catalyst loading. With this catalyst a high turnover frequency (TOF) of 55,000 h^−1^ in the hydrogenation of acetophenone was achieved. Replacement of acetate bidentate ligand in the catalyst with bidentate diethylamine improved the catalytic activity of the complex. The novel catalyst afforded 1-phenylethanol in 86% in 1 h. The TOF of the reaction was also increased up to 140,000 h^−1^.

**Scheme 11:**
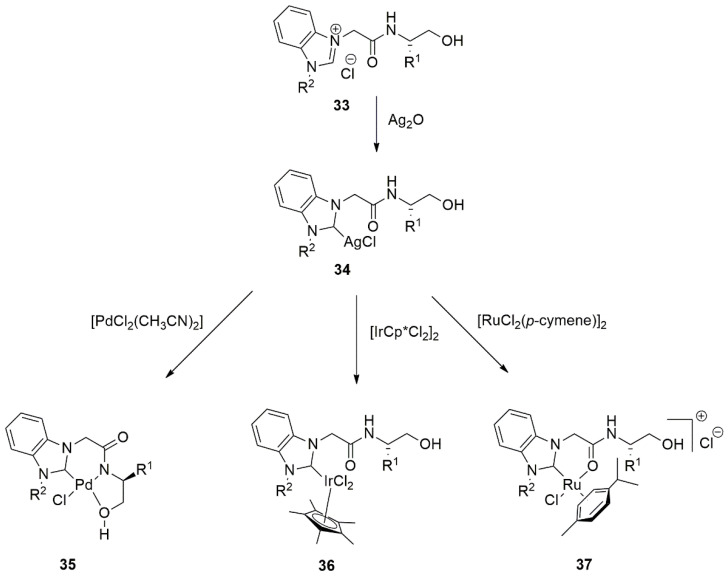
Amide functionalized NHC ligand and its metal complexation [29]. Cp* = Me_5_-Cp.

The synthesis of a couple of Ru(II) complexes containing N-heterocyclic carbenes functionalized with a secondary oxygen donor was reported by Sakaguchi et al. [29]. As shown in Scheme 11, the synthesis of ruthenium complex was carried out by transmetalation of silver complex of hydroxy-amide-functionalized benzimidazolium salt. 

Utilizing the same ligand system, this group previously reported the synthesis of palladium and iridium complexes (Scheme 9) [119]. With palladium metal, the ligand lost an HCl unit, via activation of amide N-H bond, and turned into a neutral metal complex, the similar procedure with an iridium metal precursor led to the monodentate coordination of NHC ligand. As shown in Scheme 11, by treating silver complex of the ligand with [RuCl_2_(pcymene)]_2_] as a ruthenium precursor, a cationic complex is produced in which NHC ligand coordinates as a chelating ligand. The complex **37** (R^1^ = *i*Pr, R^2^ = Me), showed low catalytic activity and enantioselectivity (19% and 31%, respectively) in the TH of acetophenone to the corresponding chiral alcohol. 

**Scheme 12:**
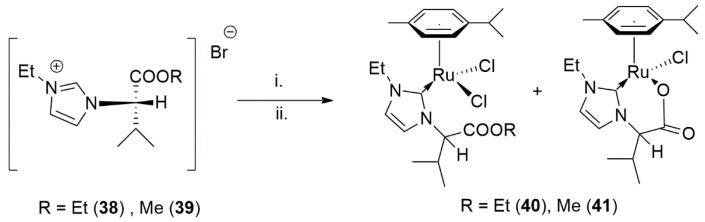
Different coordination motifs of NHC-carboxylate [123]. Reagents and conditions: (i) Ag_2_O, CH_2_Cl_2_, RT, 18 h; (ii) [(η^6^-*p*-cymene)RuCl_2_]_2_, CH_2_Cl_2_, RT, 24 h.

Using an imidazole-based NHC ligand with carboxylate tether, Foley et al. in 2013 developed a similar tetrahedral Ru(II) complex that was highly active in the catalytic TH of ketones [123]. As shown in Scheme 12, the naturally occurring and enantiopure amino acid L-valin was employed as a substituent on imidazole ring, acting as a secondary donor site after transmetalation and coordination of NHC to the ruthenium center. The hydrolysis of carboxylic acid in the reaction medium, perhaps via water generated during the formation of silver-NHC complex, led to the free hydroxy acid and its subsequent coordination to the metal center. Both metal complexes with monodentate and chelating NHC ligands (**38** and **39** in Scheme 12) catalyzed TH of acetophenone with almost quantitative yield of the alcohol product after 6 h. 

**Scheme 13:**
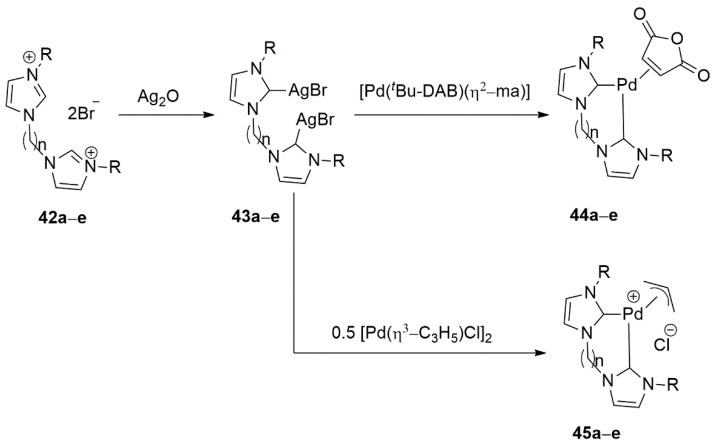
Bis-NHC and its palladium complexes [124]. *t*Bu-DAB: 1,4-di(tert-butyl)-1,4-diaza-1,3-butadiene. **a**: R = 2,4,6-Me_3_C_6_H_2_, n = 1; **b**: R =2,4,6-Me_3_C_6_H_2_, n = 2; **c**: R = Me, n = 1; **d**: R = Bn, n = 1; **e**: R = *t*Bu, n = 1.

Because of structural tunability which allows adjustments in their catalytic properties, bis(N-heterocyclic carbenes), bis-NHCs, gained lots of interest along other bidentate NHC ligands. Elsevier et al. and Hwang et al. in 2013 separately introduced this type of ligands for the development of Pd(0)-, Pd(II)-, and Ru(II)-based catalysts for hydrogenation reactions [124,125]. In the majority of cases, the synthesis of proligand salts is carried out by reacting a mono-substituted imidazole with dihaloalkanes at elevated temperatures. While in some cases direct metalation of proligands furnished the desired product, in the above example transmetalation reaction with silver complexes was required to achieve well-defined complexes. Summary of the palladation procedure is shown in Scheme 13. An interesting feature of this ligand system is an easy manipulation of the resulting metal complex via changing substituents on imidazole nitrogen or by changing the type of spacer within bis-NHC proligand. Employing different combinations of this ligand with palladium precursors, a handful of Pd(0) and Pd(II) complexes were synthesized [124]. 

These complexes were utilized as catalyst in the semihydrogenation of 1-phenyl-1-propyne, and all proved to be inactive when a non H_2_ gas source is used for the hydrogenation. On the other hand, **44a**, a Pd(0) complex showed high activity of 99% conversion and selectivity of up to 89% in the same semihydrogenation reaction of 1-phenyl-1-propyne using H_2_ gas, favoring mostly Z-alkene over E-isomer. 

**Scheme 14:**
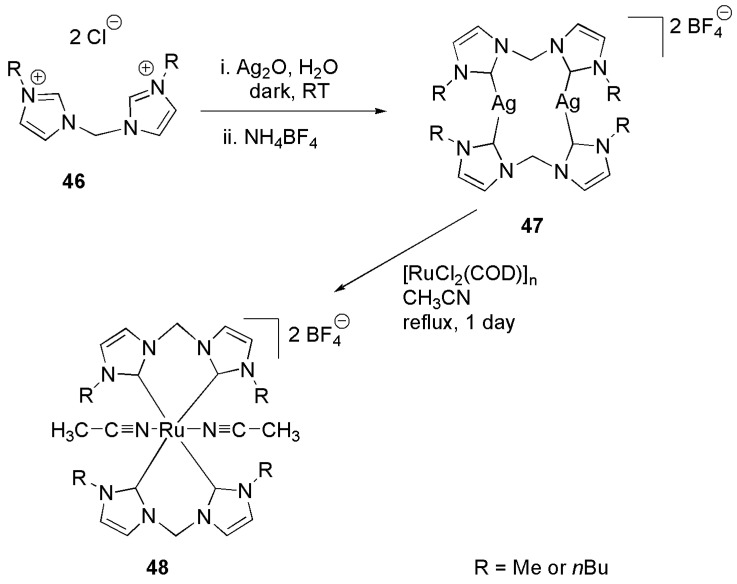
Bis-NHC and its ruthenium complexes [125].

A similar bis-NHC ligand with a methylene spacer was employed to synthesize octahedral Ru(II) complexes in which a couple of these ligands and solvent molecules are coordinated to metal center, Scheme 14 [125]. 1.0 mol% of **48** together with sodium hydroxide was sufficient for a complete TH of acetophenone in *i*PrOH. 

An identical bis-NHC ligand with ethyl substituents on heterocyclic nitrogen and its Ru(II) complex was reported by Papish et al. in 2013 [126]. The highest catalytic TH activity was observed over 24 h. 

**Figure 4:**
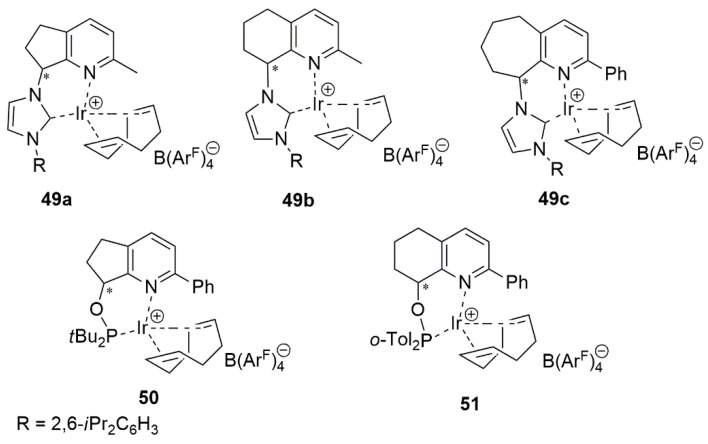
NHC-pyridine type chelating ligands and their iridium complexes [127].

Pfaltz et al. introduced a library of bidentate NHC-pyridine type chelating ligands and their iridium complexes (**49a**–**c**) for catalytic asymmetric hydrogenation of olefins [127], Figure 4. The use of these complexes permitted to avoid acid-induced side product formation during catalytic asymmetric hydrogenation of enol-ethers which occurred using related N,P ligated (pyridine phosphinite) Ir complexes **50** and **51**. The formation of less acidic iridium hydride intermediates during hydrogenation was determined to be the main feature of the NHC-pyridine complexes, which caused successful hydrogenation of acid-sensitive substrates. 

**Scheme 15:**
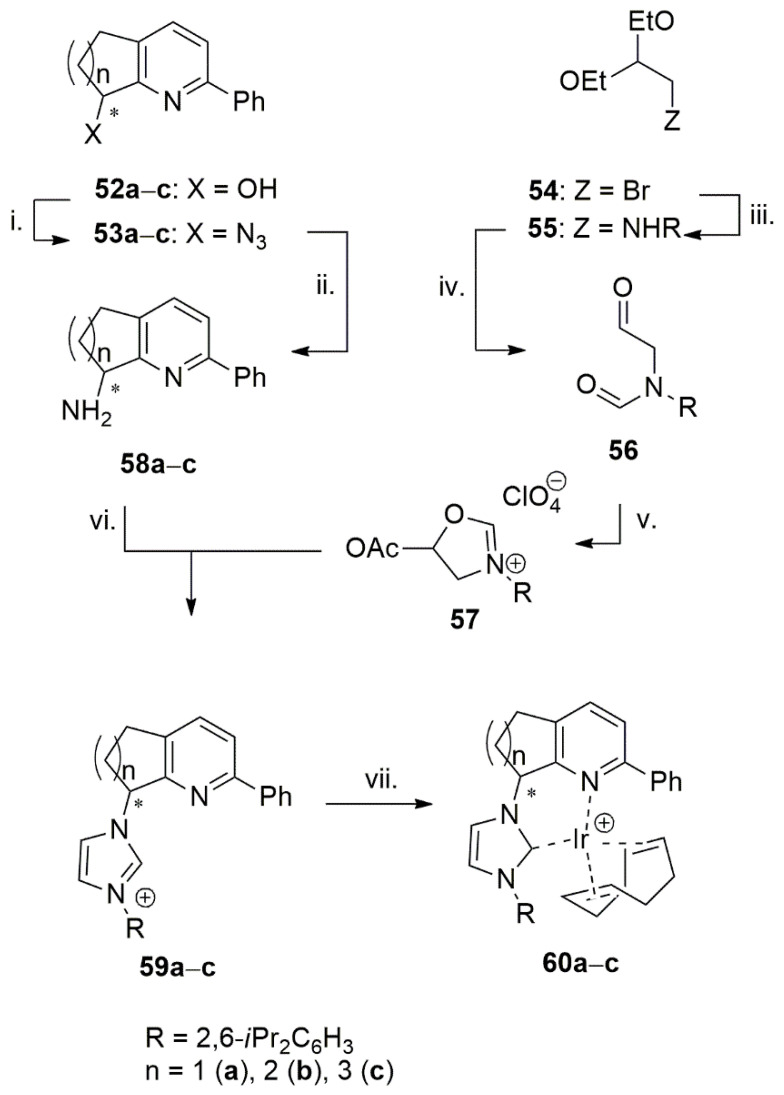
Multistep synthesis of bidentate NHC-pyridine type ligands and their Ir complexes [127]. (i) MsCl, Et_3_N, DMAP (cat.), NaN_3_, THF, 0 °C, 20 min, then DMSO, RT, 2.5 h (quant.). (ii) Pd/C, H_2_, RT, 4 h, EtOH (quant.). (iii) *n*BuLi, RNH_2_, THF (95%). (iv) HCO_2_H, Ac_2_O, THF (87%). (v) Ac_2_O, HClO_4_ (aq.) (75–80%). (vi) Toluene; Et_2_O, HClO_4_ (aq.) (41–65%). (vii) LiO*t*Bu, THF, [{Ir-(cod)Cl}_2_], RT, 2 h, then NaBArF, CH_2_Cl_2_, 30 min (44–72%). DMAP = 4-(*N*,*N*-dimethylamino)pyridine, DMSO = dimethylsulfoxide, Ms = methanesulfonyl.

The multistep synthesis of bidentate NHC-pyridine ligands, **59a**–**c** (Scheme 15), involves condensation reaction of amines **58a**–**c** with **57**. Compound **57** is synthesized in three steps starting from dietoxyethyl substituted aniline by its subsequent oxidation with formic acid and acetic anhydride [127]. The resulting iridium complexes, **60a**–**c**, with chelate NHC-pyridine ligands were used in catalytic asymmetric hydrogenation reactions of selected alkenes. **60a** and **60b** performed better than **60c** under 1 mol% catalytst loading and 50 bar of H_2_ gas at room temperature. 

A rare investigation on the influence of wingtip type (phenyl or pyridyl) on the cyclometallation reaction of a bidentate NHC ligand with Ru and Ir precursors appeared in 2014 [128]. The resulting Ir(III) and Ru(II) complexes showed different electronic and catalytic properties in catalytic TH of acetophenone. 

**Scheme 16:**
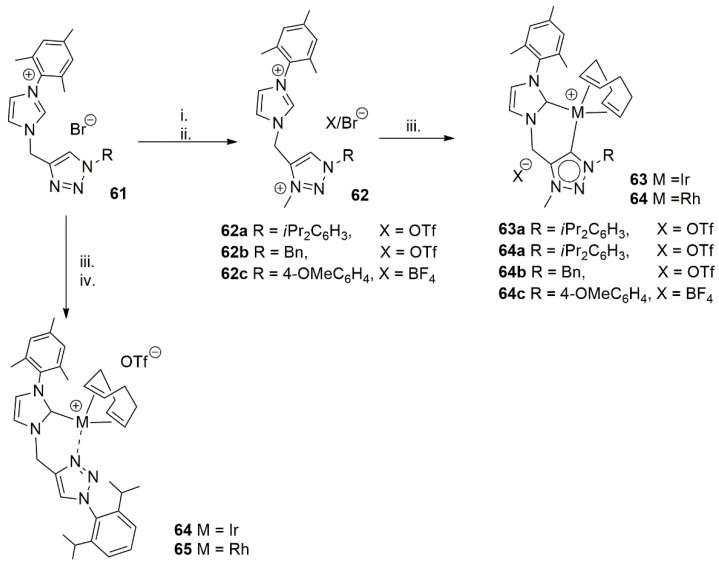
Synthesis of Ir(I) and Rh(I) complexes using bidentate NHC-N donors [129]. Reagents and conditions: (i) MeOTf, DCM, −78 °C → RT, (ii) Me_3_O·BF_4_, DCM, (iii) 1. NaH, [M(cod)Cl]_2_, MeOH; 2. L, 50 °C, (iv) 1. KO*t*Bu, [M(cod)Cl]_2_ in THF; 2. AgOTf, DCM.

Continuing their research on bidentate NHC ligands, the group of Elsevier in 2014 reported the synthesis of a novel heteroditopic NHC ligand to support rhodium and iridium as chelating ligands [129]. As shown in Scheme 16, depending on the presence or absence of substituent on N1 atom of triazole ring, both di-NHC and NHC-N(triazolyl)-supported metal complexes were achieved. 

The catalytic activity of **64** and **65** was tested in TH of acetophenone under standard condition of basic ^i^PrOH at refluxing point of this solvent. Although the results are not comparable to the previously reported highly efficient catalysts, with TOFs of up to 50,000, the outcome of catalytic activity of these complexes showed that the bis-NHC donor complexes are more active than that of the NHC-N system, perhaps via more facile formation of metal-hydride intermediate in bis-NHC systems. 

In the same year, Albrecht et al. reported the synthesis of triazolylidene ruthenium complexes with different C, O or N donor substituents. The efficacy of these Ru(II) complexes were investigated in alcohol oxidation and carbonyl or olefin TH reactions. The results revealed that donor functional groups on the triazolylidene moiety could influence catalytic activity of the ruthenium complexes [130]. 

**Scheme 17:**
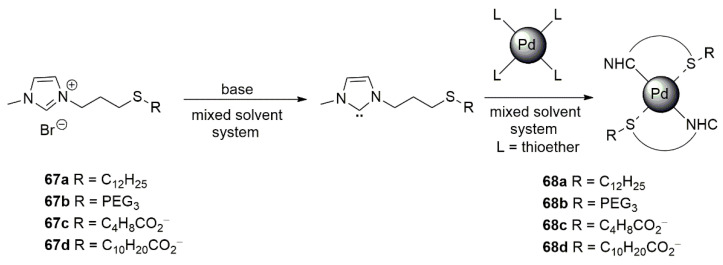
Pd nanoparticles supported with NHC-sulfur ligands [131].

An interesting application for the less explored NHC-Sulfur donor ligands appeared in 2015, in which Glorius et al. prepared four different bidentate NHC-thioether ligands, which supported palladium nanoparticles for the hydrogenation of selected terminal and internal olefines, as shown in Scheme 17 [131]. 

**Scheme 18:**
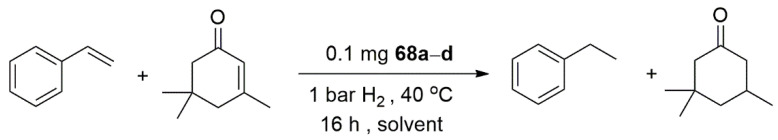
Hydrogenation of styrene with Pd nanoparticles [131].

The highly stable palladium nanoparticles showed high chemoselectivity in the hydrogenation of styrene. In comparison to **67a**,**c**,**d**, **67b** showed an overall better performance in the styrene hydrogenation in both polar and nonpolar solvents. In contrast to the palladium nanoparticles stabilized on carbon substrates, the catalytic system developed by this group showed no activity in the hydrogenation of internal olefin isophorone, shown in Scheme 18. 

**Scheme 19:**
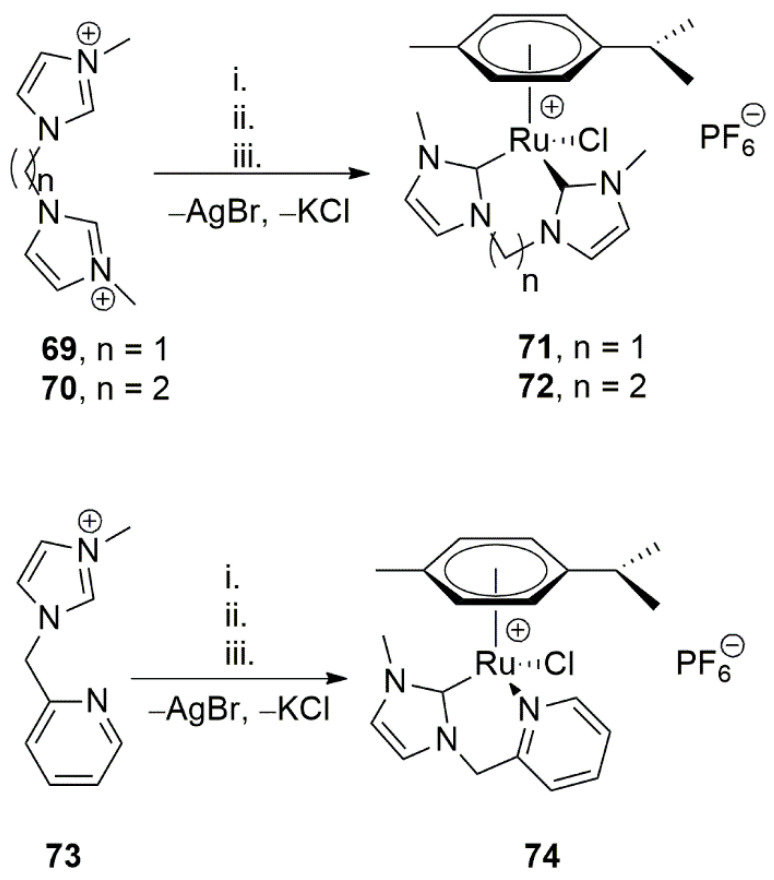
Bidentate NHC ligands and their ruthenium complexes [132]. Reagents and conditions: (i) Ag_2_O, solvent; (ii) 0.5[RuCl_2_(*p*-cymene)]_2_; (iii) KPF_6_, solvent.

Research on Ru(II) complexes with *p*-cymene and a bidentate NHC ligand or an NHC ligand with a secondary N-donor were reported by Stubbs et al. in 2016 [132]. The general structure of ligands and the resulting Ru(II) complexes are shown in Scheme 19. Using these complexes, the hydrogenation of levulinic acid was investigated. 

**Scheme 20:**
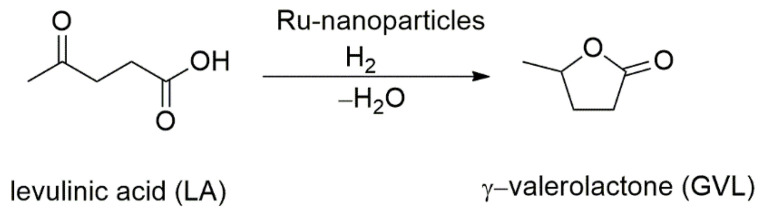
Conversion of levulinic acid to lactone [132].

A comparison with that of monodentate NHC ligands was carried out, and the results showed these metal complexes formed metal nanoparticles which turned out to be highly active in levulinic acid conversion to γ-valerolactone [132], Scheme 20. Complex **71** showed higher acivity than that of **72** and **74**. The conversion of 96% to GVL was achieved with 0.1 mol% of this catalyst in 160 min. 

*Ortho*-metalation of an aryl substituent on the imidazole or triazole was employed as a powerful method to synthesize NHC-Ru(II) complexes suitable for catalytic hydrogenation reactions [133].

**Figure 5:**
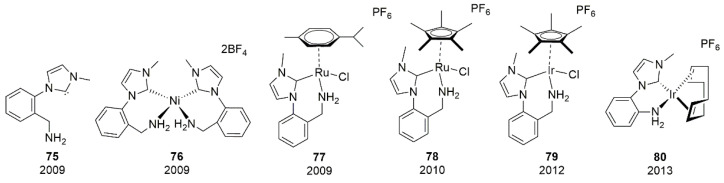
Metal complexes of NHC-NH_2_ chelates [134,135,136,137,138,139].

A series of reports regarding a primary amine tethered NHC ligand **75** (Figure 5) appeared within 2009 and 2013 [134,135,136,137,138,139]. Despite the structural simplicity of these bidentate ligands and synthetic simplicity, these achiral NHC-NH_2_ donor chelates proved to be excellent ligands for nickel, ruthenium or iridium catalyzed hydrogenation of ketones and imines.

**Figure 6:**
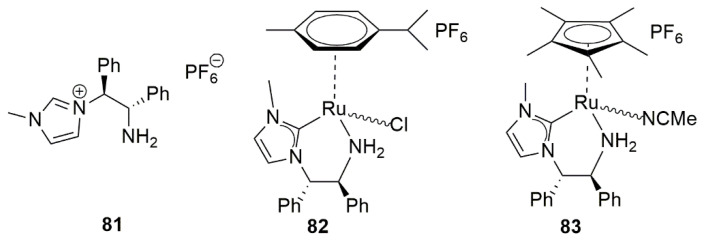
Chiral NHC-NH_2_ ligand **81** and its ruthenium complexes **82** and **83** [31,140].

An outstanding case among this particular class of ligands is the chiral proligand **81** [31], depicted in Figure 6 along with two ruthenium complexes (**82**, **83**) [140]. 

**Scheme 21:**
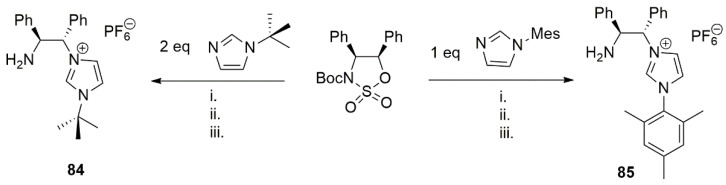
Synthesis of chiral NHC-NH_2_ ligands **84** and **85** [30]. Reagents and conditions to the left: (i) toluene, reflux 1 h; (ii) HCl(aq); (iii) Na_2_CO_3_, KPF_6_; to the right: (i) CH_3_CN, reflux 1 h; (ii) HCl(aq); (iii) Na_2_CO_3_, KPF_6_.

These asymmetric enantiopure proligands were utilized to prepare transition metal catalysts based on ruthenium, iridium, rhodium, and copper ions. Analogous chiral NHC-NH_2_ ligands featuring bulky substituents on five membered N-heterocycle were reported in 2018 by Morris et al. [30]. They were achieved by reaction of chiral tert-butyloxycarbonyl (BOC group) sulfamidate with either mesityl-imidazole or tert-butylimidazole and subsequent acid catalyzed deprotection and base neutralization (Scheme 21).

Silver oxide, due to lack of enough basicity in this case, is not sufficient to deprotonate imidazolium salt, **84**; therefore, standard silver oxide transmetalation route could not be applied. Instead, silver iodide in the presence of a stronger base such as potassium bis(trimethylsilyl)amide provided the desired silver complex. However, the silver complex obtained using this procedure was light and water sensitive and therefore unsuitable for transmetalation reactions. Utilizing a procedure developed by Song et al. [141], reaction of [Ir(O*t*Bu)(cod)]_2_ with imidazolium salts **84** was also carried out, resulting in a complex where two [IrCl(cod)] fragments are bridged by the amine-tethered NHC ligand [30].

**Scheme 22:**
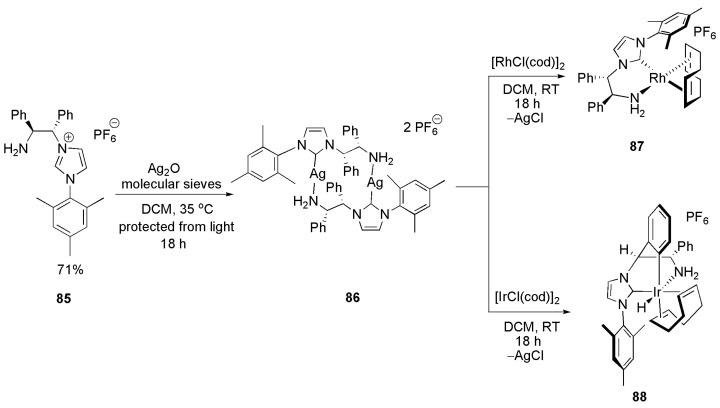
Rhodium and iridium complexes of bidentate NHC-NH_2_ ligand [30].

While transmetalation of imidazoluim salt **84** seemed challenging, in particular, when weakly chelating primary amine group remains uncoordinated, less basic proligand **85** with mesityl substituent proved to be a better chelating ligand. Reaction of **85** with silver oxide afforded the binuclear silver complex **86** (Scheme 22). Complex **86** showed to be a better transmetalation ligand than **84**. Despite using structurally analogous iridium and rhodium precursors, their transmetalation products, **87** and **88**, are different. While the expected product was obtained after transmetalation with [RhCl(cod)]_2_, an *ortho*-metalation of a phenyl ring on amine side chain and subsequent formation of iridium(III) metal complex was observed under transmetalation of **86** with [IrCl(cod)]_2_.

Complex **88** was an effective catalyst in reducing aryl-alkyl ketones, although enantioselectivity was low. This catalyst was also effective in the hydrogenation of derivatives of acetophenone with moderate enantioselectivity. 

In the past 2–3 years, several interesting reports regarding application of functionalized NHC ligands in the hydrogenation reactions appeared in the literature [142,143,144]. These advances, a result of a clever choice of a *3d* transition metal ion and employing innovative ligand design procedures, allowed catalytic hydrogenation of olefines and carbonyl compounds to occur in the ppm level of catalyst loadings. These progresses perhaps signal future directions in the catalyst design using NHC ligands to replace previously known ones, based on precious metals such as ruthenium- or iridium-based catalysts, which are well known in the field of catalytic hydrogenation reactions. 

**Scheme 23:**
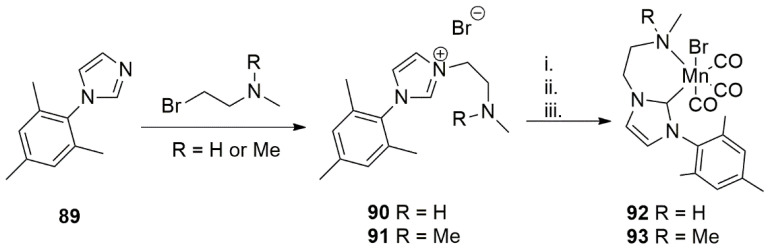
Mn(I)-NHC-based hydrogenation catalyst [145]. Reagents and conditions: (i) KPF_6_ in basic H_2_O/DCM; (ii) KN(SiMe_3_)_2_ in THF; (iii) Mn(CO)_5_Br.Using Mn(I)-NHC complex **92** a high TON of at least 17,000 was achieved in the TH reaction of acetophenone with 94% yield. Besides, a low catalyst loading in this case was close to catalysts based on Ru and Ir metals [145].

In 2019, Pidko et al. reported the synthesis of a novel Mn(I) carbonyl complexes bearing a functionalized NHC ligand. N-alkylation and subsequent metalation of functionalized ligand yielded in the octahedral Mn(I) complexes, as shown in Scheme 23 [145]. 

**Scheme 24:**
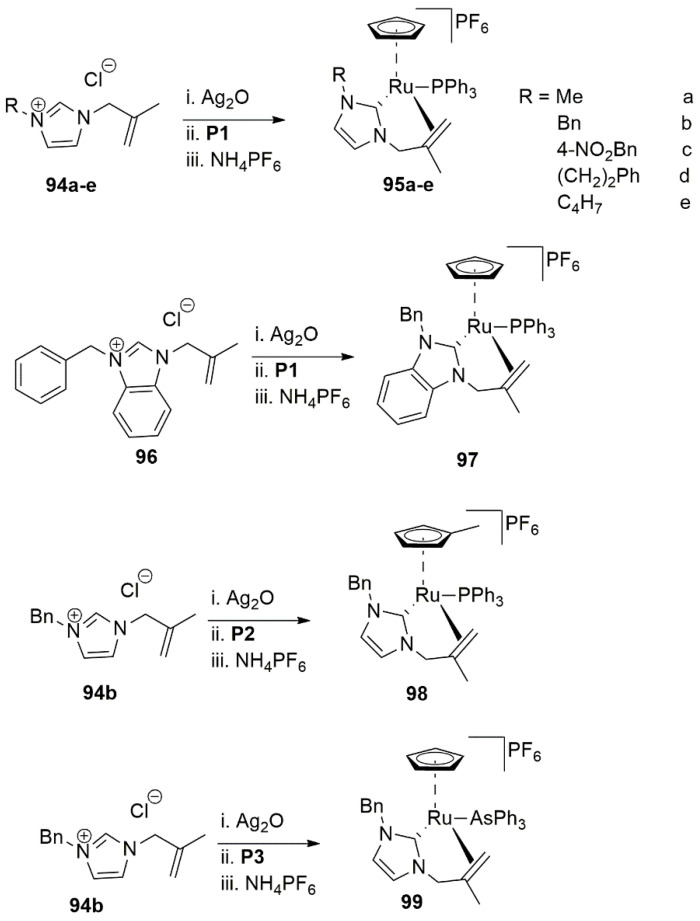
NHC-olefin ligands and their Ru(II) complexes [146]. **P1**–**3** are defined in the text.

A rare example of an NHC-alkene ligand which acts a supporting bidentate ligand in the catalytic hydrogenation reaction was reported in 2019 by Landman et al. [146]. The olefin functionalized NHC was obtained by quaternization of an N-substituted imidazole using 3-chloro-2-methyl propene. The ruthenation of the ligands are shown in Scheme 24, which follows the traditional transmetalation of the tetrahedral ruthenium complexes of general formula [(η^5^-C_5_H_4_R′)RuCl(EPh_3_)_2_] (R′ = H, E = P, **P1**; R′ = Me, E = P, **P2**; R′ = H, E = As, **P3**) with in situ generated silver-NHC complex.

**95a**–**e** and **97**–**99** were tested in the tandem hydrogenation-epoxidation reactions of derivatives of phenacyl bromide. All, except **95c**, showed moderate to good activity with reaction yields in the 36% and 69% range. 

**Scheme 25:**
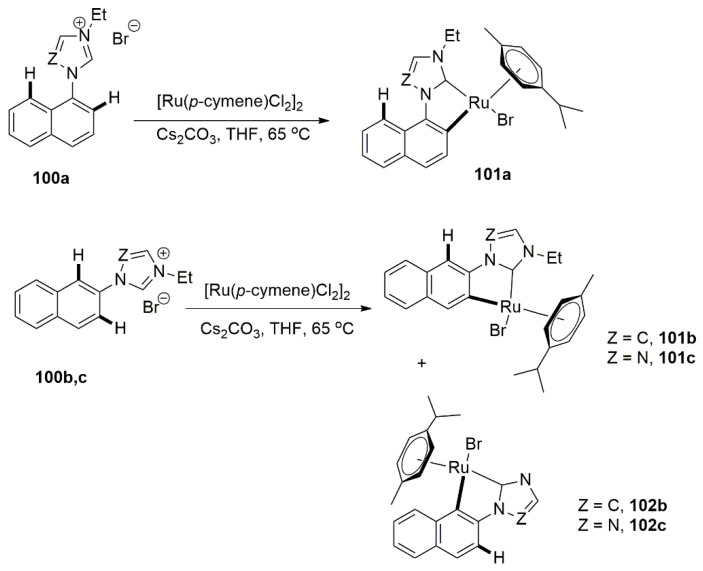
Ru(II) complexes with NHC-C ligands [147].

Recently, Rit et al. published interesting research concerning *ortho*-metalation of naphthyl substituent for catalytic reactions [147,148]. As shown in Scheme 25, the substitution pattern at the naphthyl as well as NHC coordination modes allowed the preparation of a small collection of Ru(II) complexes with different steric and electronic properties. 

Out of five metal complexes, **101b** proved to be an ideal catalyst in the TH of acetophenone in a basic *i*PrOH. Under optimized reaction conditions, hydrogenation of a series of aromatic and aliphatic ketones were carried out with high reactions rates (up to 97% in 2 h, in some cases) at low catalyst loading (0.1 mol%). 

**Scheme 26:**
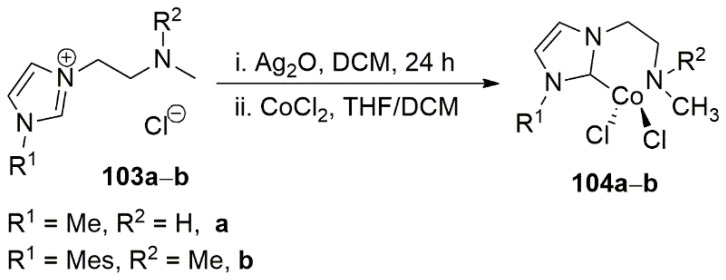
Novel Co complexes with NHC-N donor ligands [149].

Being a congener of the catalytically important precious metals rhodium and iridium, cobalt has also attracted a lot of attention mainly because of its lesser toxicity and higher natural abundance. Well-defined complexes of cobalt with amine, phosphine and in recent years with NHC ligands were applied as catalyst in the hydrogenation of a variety of unsaturated organic functional groups such as olefines, ketones, aldehydes, imines, nitriles, etc. In 2020, Liu et al. published the utility of a simple bidentate NHC ligand to support Co(II) for the hydrogenation of alkenes using H_2_ gas. Tetrahedral cobalt complexes in Scheme 26 showed high catalytic activity in the hydrogenation of simple and multisubstituted olefines [149].

For example, the trisubstituted cyclic enamine was transformed into the target product in 95% yield. Also, this simple catalytic system showed high chemoselectivity in the hydrogenation of the double bond of α,β-unsaturated carbonyl compounds, yielding in good to excellent yields. This is in sharp contrast with hydrogenation of identical substrates with well-known pincer PNP cobalt catalysts by which carbonyl groups were hydrogenated instead of olefin double bonds.

**Figure 7:**
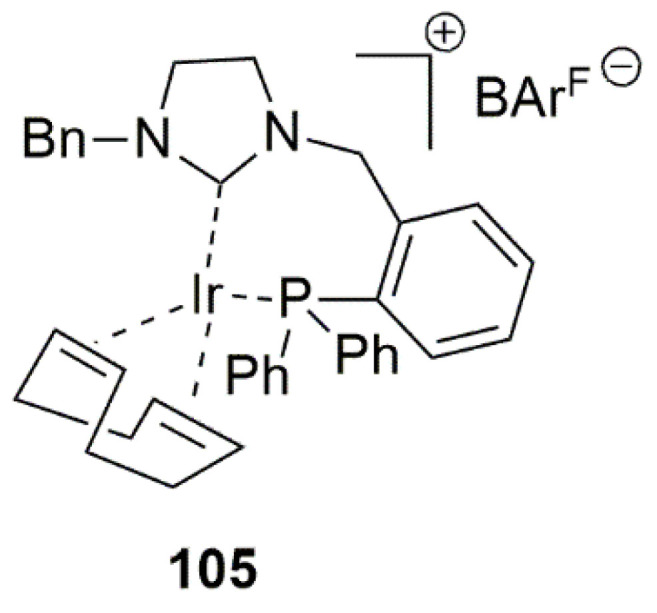
An Ir(I) complex with NHC-P donor [150].

A highly efficient hydrogenation catalyst was introduced by Anderson et al. in 2020 [150]. This group developed a novel bidentate NHC-phosphine iridium complexes that unlike previously reported cases, catalyzed complete hydrogenation of aromatic ketones in 30 min under 1 bar of H_2_ with only 1 mol% of the catalyst with high enantioselectivity. An interesting feature of this class of catalysts was that it did not require the presence of a base and that a bar of hydrogen gas was adequate for the hydrogenation. As shown in Figure 7, the catalyst **105** is composed of NHC ligand with a linked phosphine donor atom. This observation once again proves a great potential of bidentate ligands in the catalytic hydrogenation reactions. 

A mechanistic investigation of the catalytic hydrogenation cycle revealed that in this case, similarly to some previously reported hydrogenations using iridium complexes under H_2_ gas, a direct hydrogenation pathway is operative. That means the formation of iridium hydride and the migratory insertion of the hydride ligand to coordinated carbonyl substrate do occur during the catalytic cycle. 

### 2.2. Chiral Catalysis Involving Bidentate NHC Ligands

N-heterocyclic carbene ligands linked with a tether containing a heteroatom were utilized in asymmetric catalysis. Among these reactions, catalytic asymmetric hydrogenation is highly attractive due to its high atom economy and the production of optically active materials for different applications. A perspective article in this regard appeared in 2016 by Glorious et al. which describes recent advances in this field [151]. Due to tremendous effect of chiral catalysis in the advancement of procedures leading to fine chemical with a wide range of applications, selected examples from recent reports of bidentate ligands with an NHC ligand as main donor site is discussed below. These examples cover research results from a handful of scientists who contributed mostly to the advancement of this class of ligands in recent years. 

**Scheme 27:**
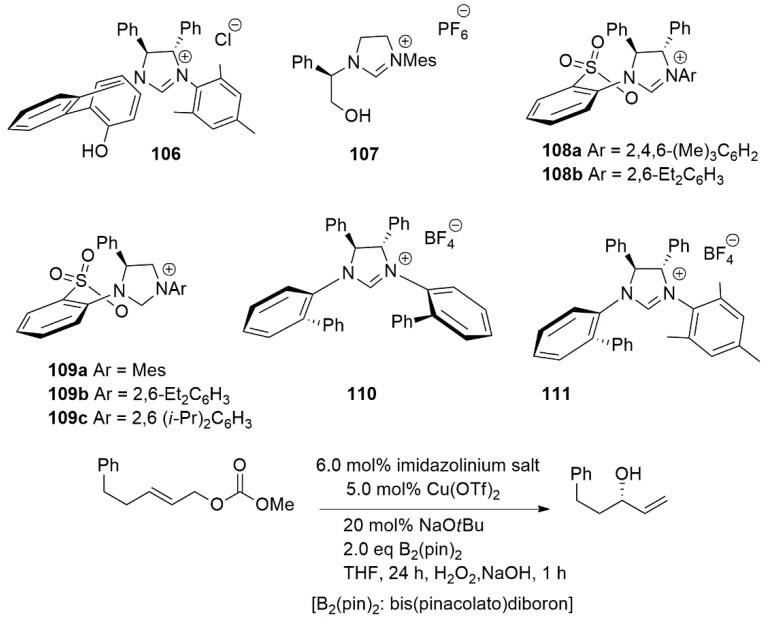
Chiral NHC ligands in allylic alcohol synthesis [101].

Allylic substitution reactions affording α-substituted allylboronates are used as nucleophiles for the addition to unsaturated substrates such as carbonyls or imines [152,153]. The synthesis of α-substituted allylboronates with tertiary and quaternary stereogenic centers was investigated using copper-phosphine complexes and earlier works in this regard were published by Ito and Sawamura [152]. In their reports, the choice of substrate was limited to the use of *Z*-allylic carbonates with linear alkyl substituents and very low enantioselectivity was obtained with *E* isomers [152]. In an effort to address this issue in the synthesis of allylboronates, Hoveyda et al. introduced a series of chiral bidentate ligands bearing NHC chelates. In situ generated chiral Cu-NHC complexes, obtained from proligands **106**–**111** (Scheme 27), catalyzed transformations with various allylic carbonates, producing allylboronates with a tertiary or a quaternary α carbon stereogenic center. Subsequent oxidation provided enantiomerically enriched allylic alcohols. The catalytic system introduced by this group performed well with *E*-disubstituted allylic carbonates as well as with trisubstituted alkyl or aryl alkenes [101]. Reaction yields in these transformations varied between 81% and 97%. High enantioselectivity (up to 98:2) was also obtained. 

The most enantioselective ligand system, among those employing the bidentate NHC-sulfonate ligands **106**–**111** (Scheme 27), proved to be the Cu-NHC complex of **109c**, bearing a di-*i*propylphenyl moiety.

Using similar strategy and employing same class of chiral Bidentate NHC ligands and their copper complexes, this group extended this methodology for the enantioselective allylic substitution involving aryl- and hetero-arylmetal reagents [77]. For Cu-catalyzed enantioselective allylic substitution (EAS) reactions of aryl-substituted vinylmetals [76].

**Scheme 28:**
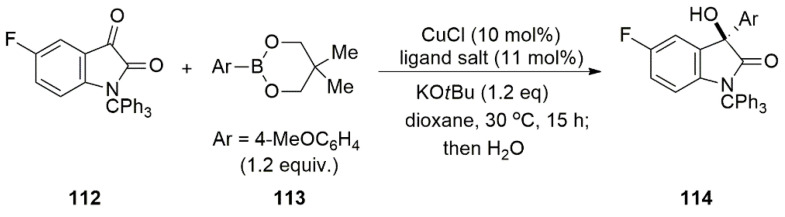
Addition of arylboronate to isatin [85].

**Figure 8:**
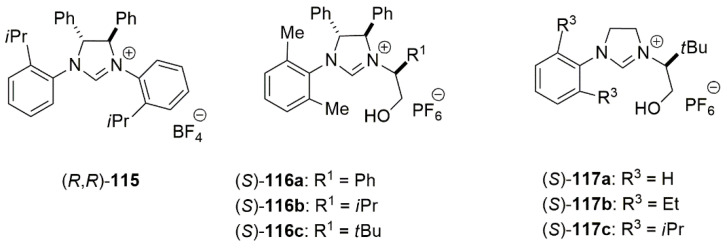
Chiral mono- and bidentate NHC-hydroxy ligands [85].

Bidentate NHC ligands bearing chiral groups were extensively employed since 2010 as cocatalysts to facilitate asymmetric catalysis. Hayashi et al. reported the synthesis of copper-catalyzed addition of isatins **112** to arylboronates **113** to give 3-aryl-3-hydroxy-2-oxindoles **114** (Scheme 28). To obtain enantiopure product, the chiral bidentate NHC ligands **115**–**117** (Figure 8) were tested [85]. The catalytic reaction in the presence of **115** with chiral center at the backbone of imidazolium salt, gave poor results, yielding in enantiomeric excess of 8%. 

Higher enantioselectivity was obtained using 2,6-dimethylphenyl substituent on the heterocycle nitrogen. The highest enantiomeric excess (88%) and reaction yield (80%) was obtained using *C*_1_-symmetric **116** salt. Other alternatives of these bidentate ligands, **117a**–**c**, did not perform well in enantioselectivity. 

**Figure 9:**
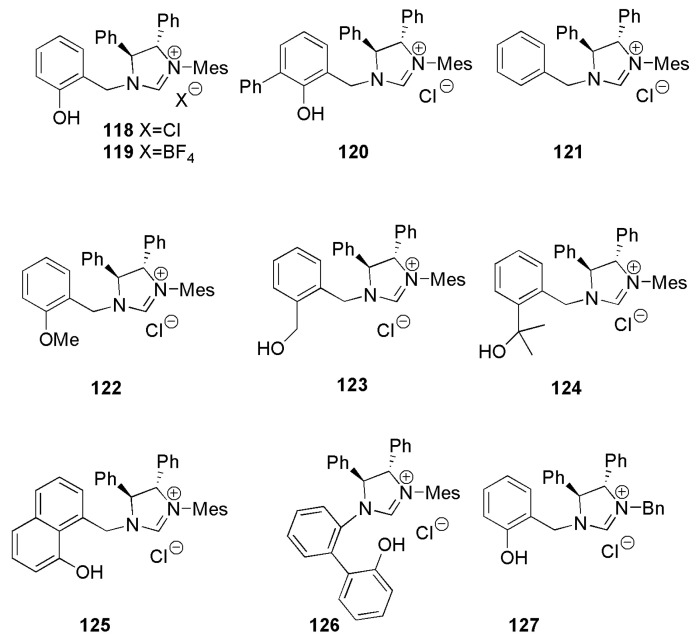
Chiral bidentate NHC-hydroxy ligands [154].

Several research groups also investigated allylic alkylation reactions using Grignard reagents since early 2000. An interesting example of a bidentate NHC ligand which does not require the presence of copper catalysts during alkylation reactions was introduced in 2010 [154]. Alexakis et al. developed the bidentate NHC ligands **118**–**127** (Figure 9) endowed with a diphenyl imidazoline core and a mesityl group on one nitrogen atom [154]. The second nitrogen atom of the heterocycle was substituted with a benzylic group. The chelating ligands **118**–**127** were used, along with Grignard reagents and a copper(II) metal source such as Cu(OTf)_2_, to catalyze asymmetric allylic alkylation (AAA) reaction. These reactions work well in diethyl ether solvent with only 1 mol% of **118**, and reactions yields close to 98%. It turned out that certain enantioselective AAA reactions did not require the presence of copper catalysts.

**Figure 10:**
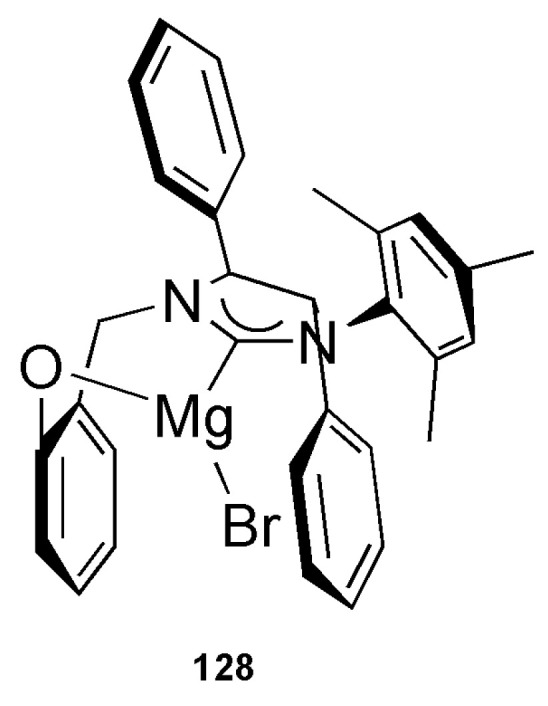
Structure of a magnesium NHC-phenoxy chelate [154].

By screening AAA reactions concerning Grignard reagents, substrate and leaving groups, it was noticed that the catalytic reactions are initiated by the magnesium NHC-phenoxy chelate **128** (Figure 10) [154].

**Figure 11:**
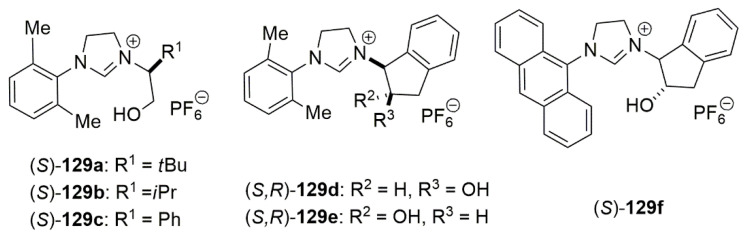
Chiral mono- and bidentate NHC ligands [156].

**Scheme 29:**
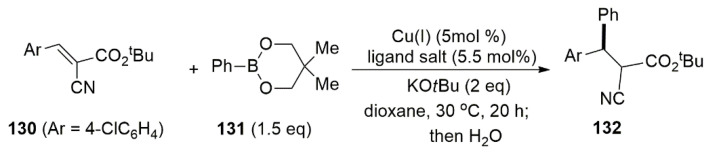
Copper catalyzed 1,4 addition of organoboronates to alkenes [156].

Carbon–carbon bond forming reactions such as 1,4-addition of organometallic reagents to α,β-unsaturated compounds employing copper catalysts are powerful methods for the synthesis of organic molecules [155]. While most methodologies, employing copper catalysts, include reactive nucleophiles such as Grignard reagents or diorganozinc compounds, with milder nucleophiles such as organoboronic compounds reports are rare. These compounds, because of ease of handling and inherent stability would be interesting targets in search for a greener approach for the 1,4-addition of organometallic reagents to α,β-unsaturated compounds. An interesting contribution in this regard was given by Hayashi et al., which employed both symmetric monodentate **129d**, **129e** and asymmetric bidentate NHC ligands **129a**–**c**, **129f** with anionic tethers (Figure 11) for enantioselective addition of organoboronates to alkylidenes (Scheme 29) [156]. 

For the catalytic asymmetric coupling reaction shown in Scheme 29, a series of bidentate NHC ligands in the presence of copper chloride or copper bromide were tested, with the best result in terms of enantioselectivity observed when (*S*)-**129f** with a bulky 9-anthryl was used. The combination of this ligand with CuBr to the formation of **132** with 92% ee and 86% yield. 

Various aryl groups from boronates and alkyls such as 1-cyclohexenyl- and methylboronates can also be incorporated effectively from the nucleophilic component into benzylidene cyanoacetate [155]. Using the same class of NHC-Cu systems, Hayashi et al. also reported the asymmetric allylic substitution of allyl phosphates with aryl- and alkenylboronates [156]. 

**Scheme 30:**
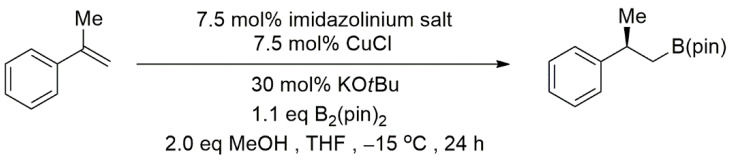
Cu-NHC catalyzed hydroboration of 1,1-disubstitued aryl olefines [106].

Highly active Cu-NHC catalyzed hydroboration of 1,1-disubstitued aryl olefines by the use of organoboronic reagents was reported in 2011 by Hoveyda et al. [106]. In this research, enantioselective hydroboration of acyclic and exocyclic 1,1-disubstituted aryl alkenes were reported, Scheme 30.

**Figure 12:**
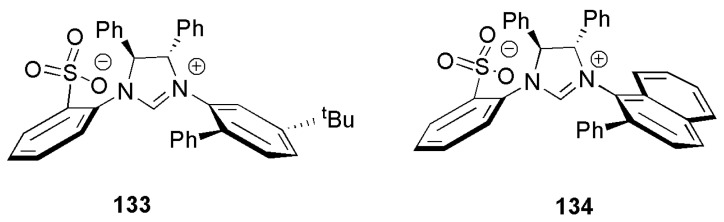
Bulky enantioselective chiral ligands in olefin hydroboration [106].

Among different imidazolium salts examined, a nonchelating aryl with tert-butyl substituent on the *meta* position (**133**) or naphthyl-substituted salt (**134**) proved to be the most enantioselective ligands, Figure 12. The product with 98% in yield and e.r. of 85.4:14.5 was achieved with ligand **134**. 

The catalytic enantioselective addition of carbon-based nucleophiles to unsaturated carbonyl compounds remains a great strategy for the generation of biologically active molecules. Recent innovative work in this area included the use of vinylmetal reagents. Check out [157,158] for catalytic enantioselective conjugate addition of vinylzirconiums and silanes. 

**Figure 13:**
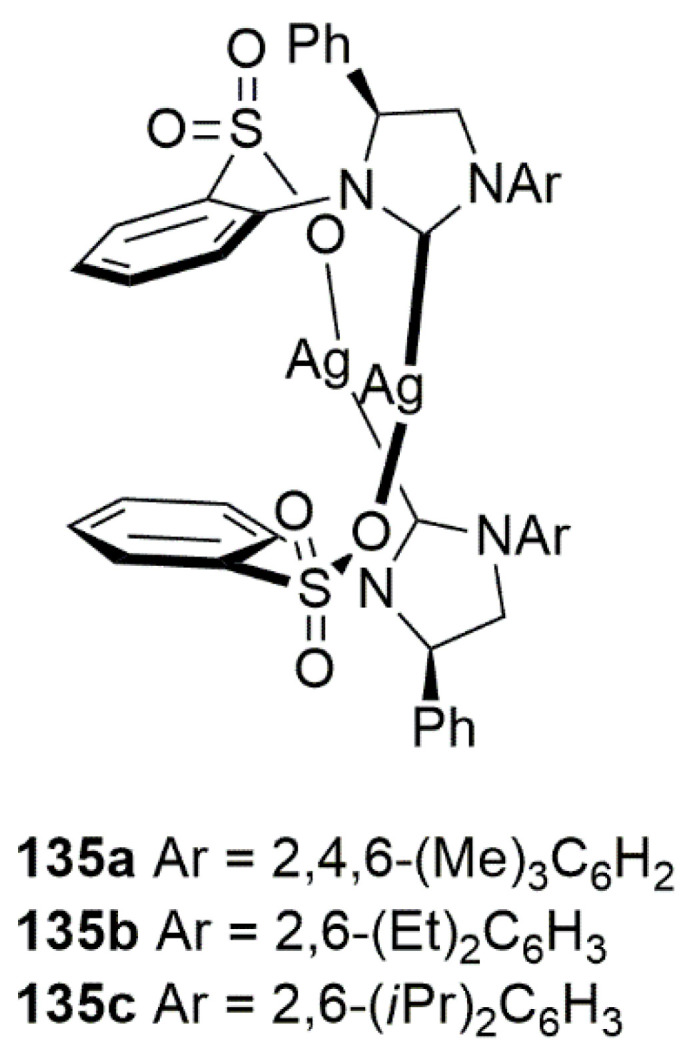
Dimeric silver complexes with NHC-sulfonate donors [90].

To address substrate and reagent scope in this area, Hoveyda et al. reported successful utilization of Cu-NHC based catalysts for the enantioselective addition of vinylaluminum reagents substituted with silicone. Among different bidentate NHC-based ligands tested for this purpose, the NHC-Cu complex formed in situ from dimeric silver complex shown in Figure 13, by addition of CuCl_2_·2H_2_O, proved to the most efficient catalyst [90]. Among three silver complexes examined for in this reaction, **135b** gave β-substituted cyclic enones in quantitative yield with enantiomeric ratio of up to 98:2. 

**Scheme 31:**
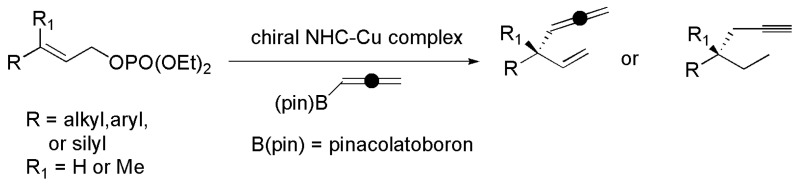
Allenylboronic acid pinacol ester addition to alkenes [102].

Hoveyda et al. reported another novel catalytic enantioselective allylic substitution reaction using analogous bidentate NHC-sulfonate ligands. In this case, allenylboronic acid pinacol ester addition to allylic phosphates could potentially lead to allenyl containing products or to a propargyl addition to allylic substrate (Scheme 31). The catalyst introduced by this group, a sulfonate-bearing chiral bidentate NHC-Cu complex, favored the production of allenyl containing products in almost quantitative yields with enantiomeric ratio of 95.5:4.5 [102].

**Figure 14:**
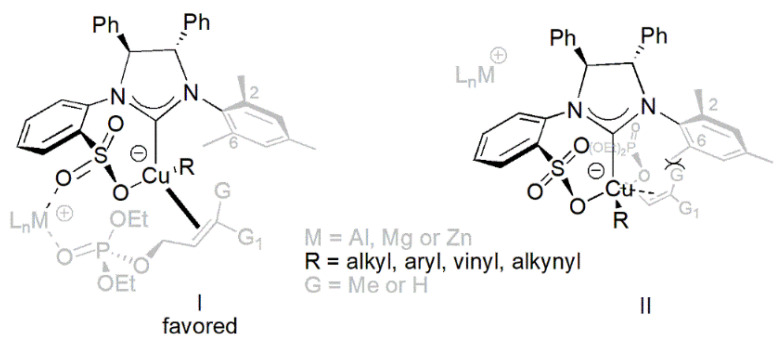
Different reactivity of organometallic reagents [102].

In enantioselective allylic substitution reactions, apart from copper salt and phosphine or NHC based ligands, organometallic (L_n_M in Figure 14) or boron-based reagents are used as co-catalysts [102]. Organometallic reagents, L_n_M, are Lewis acids and their interaction with sulfonate oxygen and phosphate unit is shown in Figure 14. Two orientations of substrate relative to Copper-R σ bond in Cu-NHC catalyzed allylic substitutions of trisubstituted allylic phosphates is shown below. Out of these two orientations, the favored orientation with less steric hindrance (I) delivers the major isomer [102].

**Scheme 32:**
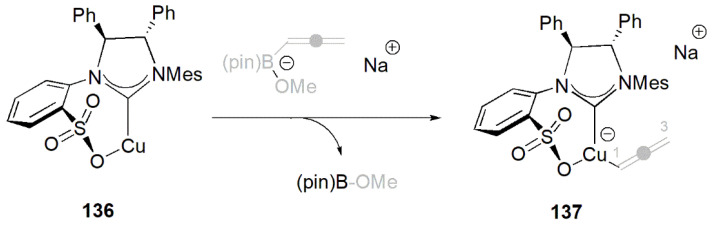
Sodium alkoxide mediated ally transfer reaction from allylboronate [102].

In contrast to organometallic cocatalysts, when allylboronate is used as reagent, the presence of a metal alkoxide, such as NaOMe, as depicted in Scheme 32, allows the formation of NHC-Cu-allene **137** via transmetalation with allenylboronate. 

**Figure 15:**
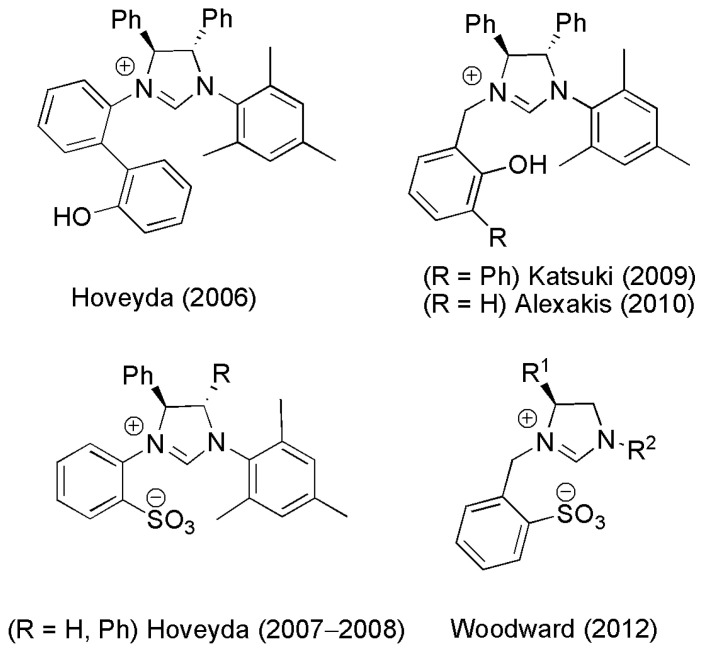
Azolium salts containing a chiral moiety [97].

Taking into account the high utility of N-heterocyclic carbene ligands and their metal complexes in a variety of organic transformations, Woodward et al. in 2012 introduced a novel method for the synthesis of bidentate N-heterocyclic carbene ligands with sulfonate donors. While Hoveyda et al. reported the synthesis and application of these sorts of ligands previously, the methodology introduced by this group dramatically reduces the number of synthetic steps. The general structure of the ligand systems introduced by this group along with previously reported analogous structures are depicted in Figure 15 [97,159,160,161,162]. 

**Scheme 33:**
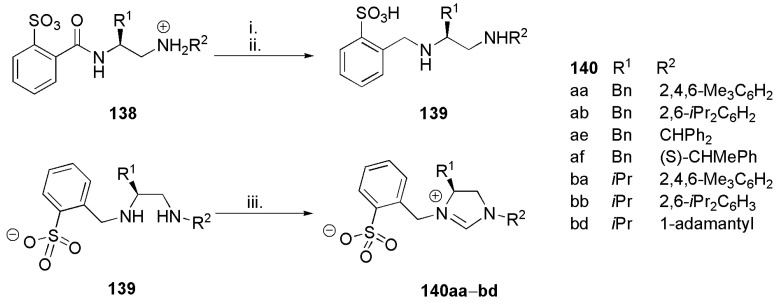
Chiral azolium salts containing a sulfonate moiety [97]. Reagent and conditions: (i) BH_3_·SMe_2_, THF, 60–90 °C, 3 d; (ii) MeOH, 90 °C, 2 h;(iii) (EtO)_3_CH, EtOH, 100 °C, 17 h.

Multistep synthesis of the bidentate NHC-sulfonate ligands **140** is shown in Scheme 33. Reduction of the amidato sulfonic acids **138** with BH_3_·SMe_2_ provided **139** which then undergoes cyclization reaction with triethylorthoformate to yield the imidazolium salt **140** [97]. In both of these reactions, many alkyl and aryl substituents, R_1_ and R_2_ are tolerated which demonstrated the strength of the methodology developed by this group.

**Scheme 34:**
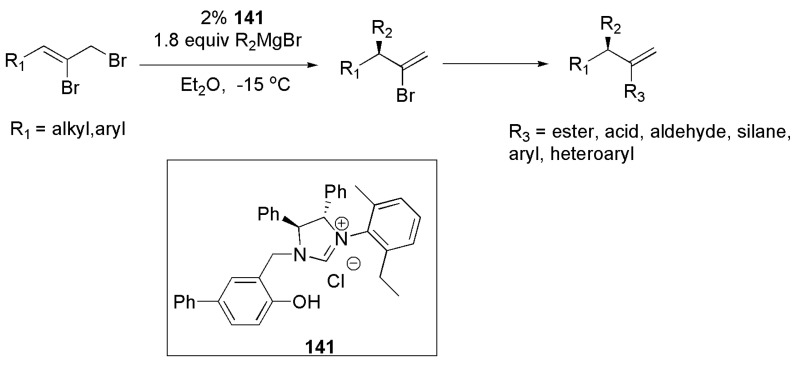
Allylic alkylation of vinyl bromide substrates [163].

Using bidentate NHC **141** and alkyl or aryl Grignard reagents, allylic alkylation of vinyl bromide substrates in a copper free reaction medium was reported by Alexakis et al. in 2012 (Scheme 34) [163]. 

Allylic alkylation of the allyl bromide in the first step, provided a functionalized intermediate which allowed for further transformation of enantiomerically enriched intermediate and the formation of 1,1-disubstituted olefines [163]. 

**Figure 16:**
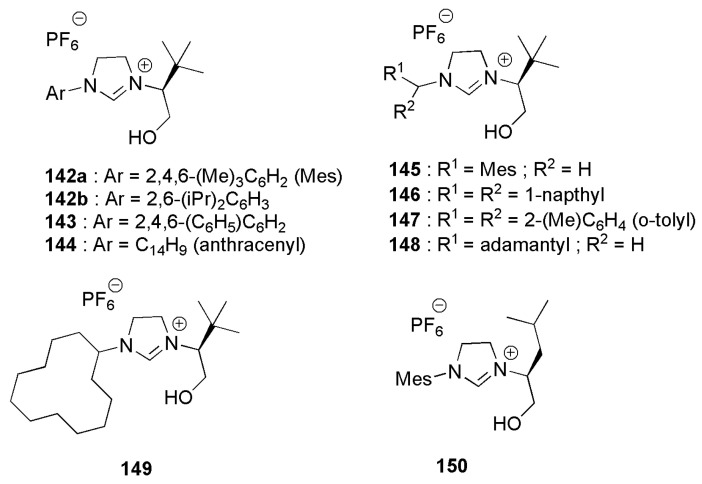
Proligands introduced for allylic alkylation of allylphosphates [164].

Maudunit et al. in 2013 demonstrated importance of bidentate proligands in the allylic alkylation of allylphosphates using Grignard reagents [164]. The chiral hydroxyalkyl bidentate imidazoline based NHC proligands introduced by this group is shown in Figure 16.

Among proligands depicted in Figure 16, **142b** showed to be the most efficient. This proligand in the presence of Cu(OTf)_2_ afforded good regio and enatioselectivity (up to 97:3 e.r.) and 99% yield with most of the tested substrates. It has been demonstrated that substituents at the N-aryl and stereogenic center play an important role in the regio- and enantioselectivity [164]. 

A multicomponent strategy for the synthesis of bidentate NHC-hydroxyalkyl or NHC-carboxyalkyl ligands was introduced by Mauduit et al. in 2014. Unlike prevous records [165,166,167], an amino alcohol or amino acid, instead of cycloalkylamines, allowed access to bulky unsymmetrical and chiral bidentate NHC ligands with tunable anionic side chain [168]. While this strategy is applicable to both amino alcohols and amino acids, the yield is negatively affected by increasing the steric demands of amino alcohols. 

**Scheme 35:**
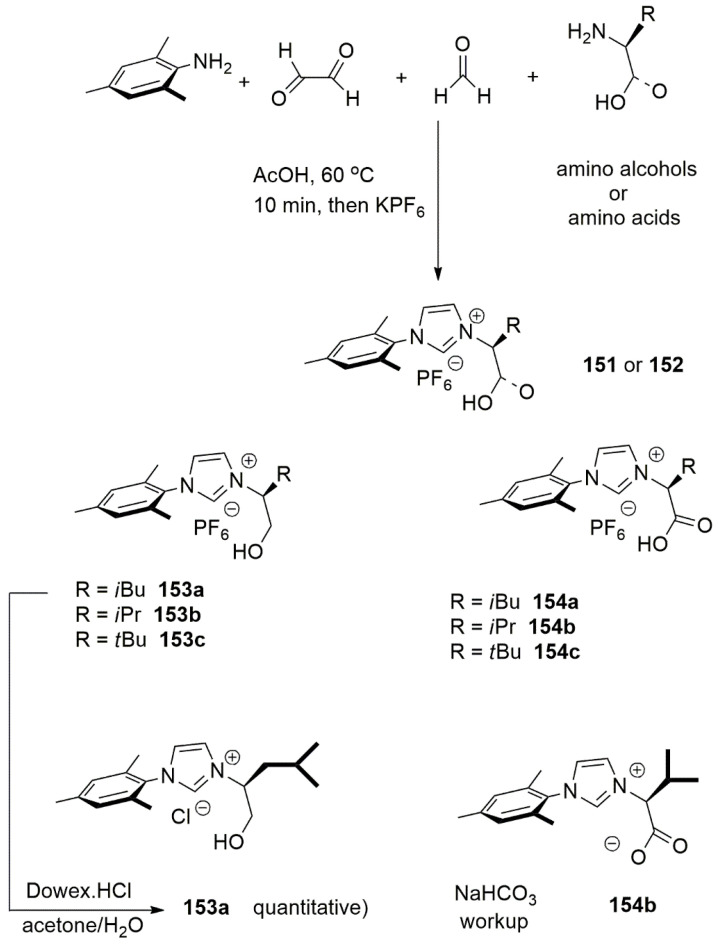
Multicomponent synthesis of chiral bidentate imidazolium salts [168].

The syntheses of chiral imidazolium salts **153**–**154**, prepared with this strategy, are shown in Scheme 35 [168].

Among these proligands with chiral side chains, **153a** showed high regio- and enantioselectivity in copper catalyzed dialkylzinc 1,4 addition with β-substituted cyclic enones (e.r. up to 99:1), 1,6-addition with cyclic dienones (e.r. up to 95.4:4.5), and allylic substitution with allyl phosphates (e.r. up to 96:4) [168].

**Scheme 36:**
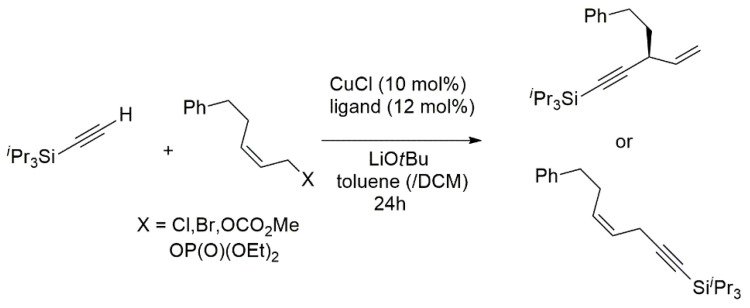
Enantioselective allylic alkylation of alkynyl nucleophiles [98].

Like allylic alkylates discussed so far, the enantioselective allylic alkylation of alkynyl nucleophiles is also a powerful strategy for organic synthesis such as chiral 1,4-enynes. In 2014, Sawamura et al. reported copper-catalyzed enantioselective allylic alkylation using terminal alkynes and primary allylic phosphates, Scheme 36 [169]. 

**Figure 17:**
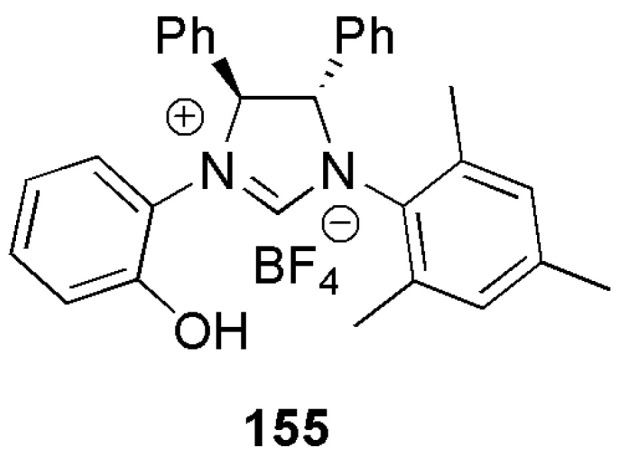
Bidentate NHC-OH ligand for enantioselective allylic alkylation [98].

While previously reported methods [98,100,170] utilized acetylide reagents such as alkynylaluminum reagents, terminal alkynes were used in this breakthrough method by this group. Out of two major regioisomers expected in this reaction, a copper N-heterocyclic carbene complex developed by this group favored branch selectivity. This observation is in sharp contrast to the previously reported reactions catalyzed by phosphine or nitrogen donor ligands. The structure of phenol type ligand with NHC core is shown in Figure 17. The high yield of 91% and branched product selectivity of 99:1 was ontained with this catalyst system. 

**Figure 18:**
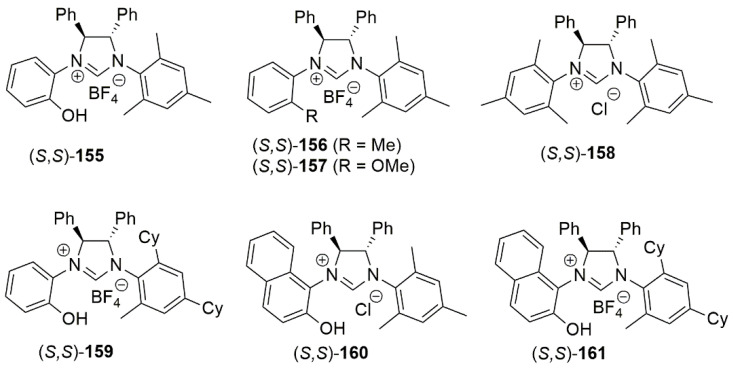
Mono- and bidentate chiral NHC ligands developed by Sawamura [81].

**Scheme 37:**
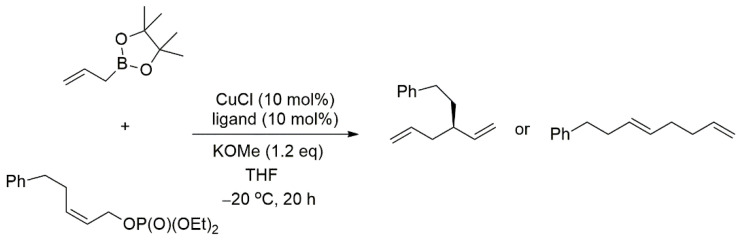
Reaction of boron pro-nucleophiles with alkynes [81].

By employing CuCl in the presence of chiral NHC ligands bearing a hydroxy group, Sawamura et al. performed enantioselective allyl–allyl coupling between allylboronates and (Z)-allylic phosphates (Scheme 37) [81]. General structures of the examined bidentate NHC-hydroxy proligands **155**–**161** are shown in Figure 18. In 2014, in a similar study, copper catalyzed allylic cross-coupling reaction of allyl chloride substrates with alkylboranes was reported [171]. 

An important feature of allyl–allyl coupling catalysts delivering chiral 1,5-diene derivatives is their functional group tolerance of acidic or basic nature. Also, *Z*-aliphatic allylic substrates including acyclic and cyclic 2-alkene-1,4-diol derivatives can be used in these catalytic reactions. While CuCl/1,3-bis(2,4,6-trimethylphenyl)imidazolinium chloride (SIMes·HCl) catalytic system gave excellent yield in initial reaction optimizations, high enantioselectivity was only achieved with ring-saturated chiral chelate NHC ligands bearing two stereogenic centers **155**–**161**, (Figure 18). 

**Figure 19:**
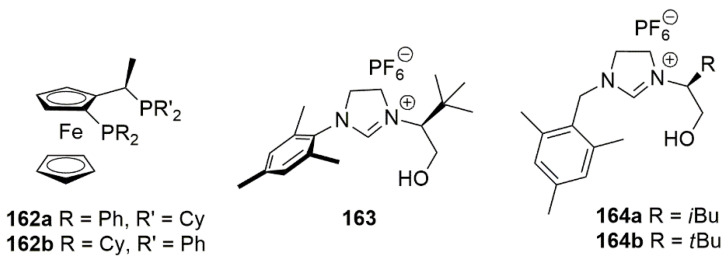
Phosphine donor and NHC donor ligands for addition of dimethyl zinc to acylimiazole [172].

A powerful method in the conjugate addition of organometallic reagents to electron deficient substrates such as Michael acceptors was disclosed by the group of Campagne et al. in 2015 [172]. Despite previous reports in this area, this group utilized the well-known organometallic reagent, dimethylzinc, for the preparation of all carbon methyl substituted chiral scaffolds with diverse applications. Among a variety of phosphine donor and NHC donor ligands which were tested, the bidentate hydroxyl-NHC, **164b**, Figure 19, was shown to be the most efficient ligand to catalyze addition of dimethyl zinc to acylimidazole in the presence of copper metal. 

**Figure 20:**
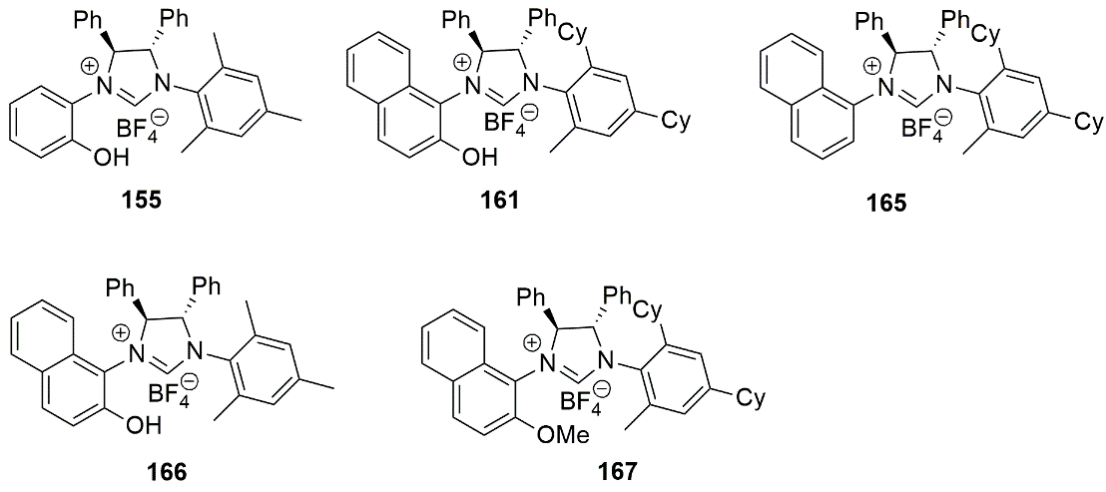
Sawamura’s enantioselective allylic alkylation ligands [174].

**Scheme 38:**
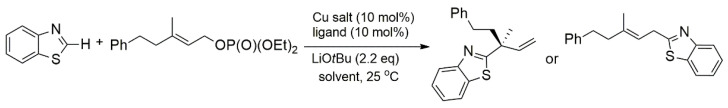
Enantioselective allylic alkylation [174].

The same group reported scope and limitations of this protocol separately in 2016 [173]. As a follow up to a previous report about copper catalyzed enantioselective allylic alkylation of terminal alkynes using bidentate NHC ligands with a phenolic side arm, another report concerning allylic alkylation was described by Sawamura et al. in 2016 [174]. A highly enantioselective azole allylic alkylation was successfully carried out by popular copper-based NHC combination [174]. Shown in Figure 20, and except one case, are bidentate NHC ligands with hydroxyl side arms that were tested in alkylation of benzothiazole (1a) with (E)-allylic phosphate, Scheme 38. 

Out of five NHC ligands, **161** with a naphtholic OH group was shown to be the most efficient in both conversion and enantioselectivity. This ligand also showed excellent branched selectivity, Scheme 38.

### 2.3. Coupling Reactions

Donor functionalized NHC ligands have also been investigated in cross-coupling reactions. Among these reactions, carbon-carbon crosscoupling reactions were targeted mostly. Huynh et al. published the synthesis and structural characterization of different forms of functionalized NHC ligands with sulfur atom as second coordination site [175]. Advances in catalytic coupling reactions using NHC ligands linked with a tether with nitrogen, sulfur, or phosphorus donor atoms as well as bis-NHC ligands from 2010 are discussed below. 

**Scheme 39:**
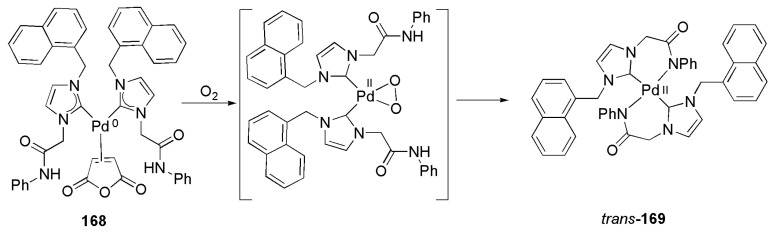
Synthesis of a Pd complex bearing a bidentate NHC-amidato ligand [176].

Lee et al. reported preparation of Pd(0)-NHC precatalyst **168** and similar structures with different substituents on the heterocyclic nitrogen for Heck type coupling reactions. However, these Pd(0) complexes, stable in the solid state, are not stable in solution or upon exposure to air [176]. Attempted crystallization in dichloromethane of **168** led to the isolation and characterization of the palladium complexes **169** bearing bidentate NHC ligands with tethered amidato functionality (Scheme 39). A proposed mechanism for the transformation of **168** into **169** involves the formation of a peroxo transient complex along with a proton exchange with amidato group, resulting in a chelate ring.

These novel NHC-amidato ligands and the resulting Pd(0) and Pd(II) complexes were successfully applied as catalysts in Heck/Heck and Suzuki/Heck coupling reactions. Pd(0) precatalysts showed higher activity in coupling reactions in ionic liquids [176].

**Scheme 40:**
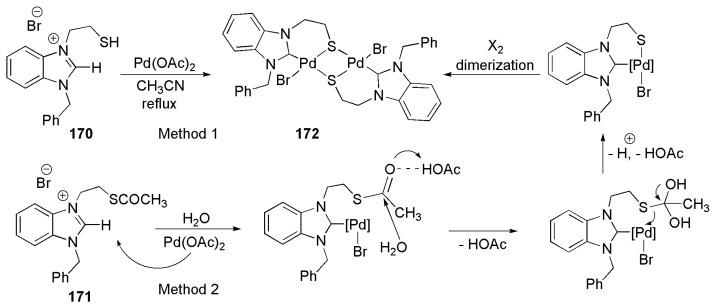
Synthesis of dimeric palladium complex with NHC-S donors [177].

Bidentate NHC ligands with sulfur donor side chain functionalities are rather rare compared to other NHC-heteroatom donors. A novel thioether functionalized NHC ligand was reported by Huynh et al. in 2010 to prepare palladium complexes [177]. As shown in Scheme 40, a binuclear complex with Pd_2_S_2_ core was prepared by the reaction of palladium acetate with either thiol-functionalized benzimidazolium salt or by the reaction of air stable benzimidazolium salt with a thioester functional group [177]. 

Complex**172** showed better catalytic activity in the Suzuki–Miyaura cross-coupling reaction over palladium complexes with phosphine donor ligands. Braunstein et al. reported a notable example of a catalytic system containing NHC ligand functionalized with thioether in 2010 [178].

**Scheme 41:**
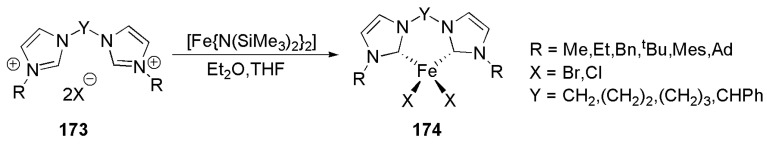
Iron(II) complex with a bis-NHC ligand for crosscoupling reactions [179].

An interesting work on iron(II)-NHC complexes preparedby direct metalation of bidentate methylene- or ethylene-bridged bis(imidazolylidene) ligands was reported in 2011 by the group of Mayer, Scheme 41 [179]. Unlike metalation with palladium, deprotonation of this type of bis-NHC ligands and subsequent metalation with chloro iron was not successful, therefore more basic bis(trimethylsilyl)amide ligand was used [179]. Also, smaller substituents on the heterocyclic nitrogen led to the formation of tetracarbene iron(II) complex.

The efficacy of these iron complexes was examined in cross-coupling reaction of *p*-tolylmagnesium bromide with bromo- or chlorocyclohexane as an example of Kumada-Curriu coupling [179]. Among tested complexes, the iron(II) dibromide complex with Y = (CH_2_)_2_ and mesityl substituents was found to be the most active catalyst. However, the activities reported in this article were generally lower than those of previously reported cases with mono-dentate ligands. 

**Scheme 42:**
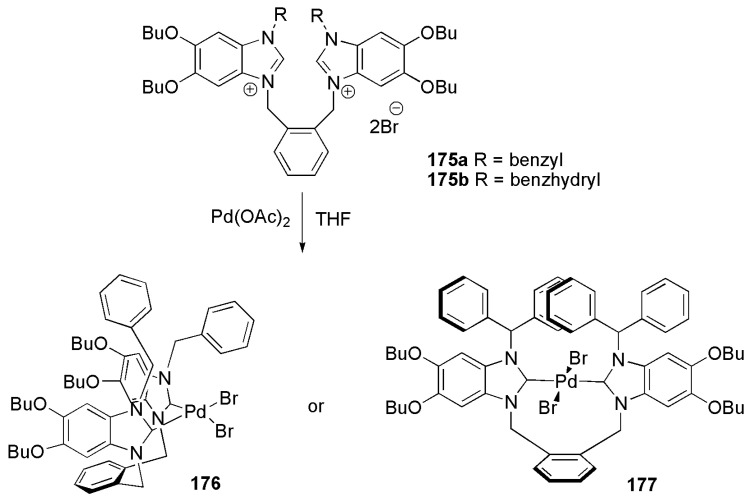
Palladium complexes with bulky bis-NHC ligands [180].

Baker et al. reported a more sophisticated bis-NHC containing structure in 2012 [180]. Shown in Scheme 42 are two palladium complexes with bulky bis-NHC ligands with an *ortho*-xylene bridging group. ^1^H and ^13^C-NMR studies confirmed *cis* and *trans* ligand arrangements in **176** and **177**, respectively. Both **176** and **177** gave moderate yields of 59% and 51% in Mizoroki–Heck cross-coupling reaction of bromobenzene and *n*-butyl acrylate. Due to better activity, **176** was also employed as catalyst in Suzuki–Miyaura cross-coupling reaction of different aryl halides with phenyl boronic acid, showing moderate to high yield with most aryl halide substrates.

**Scheme 43:**
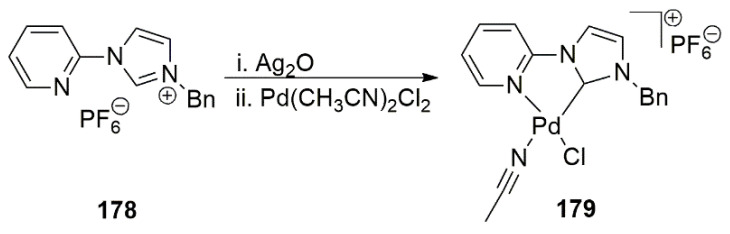
Imidazolium based NHC with pyridine side chain for Suzuki–Miyaura crosscoupling [181].

Wang et al. and Xiang et al. reported some innovative use of functionalized NHC systems and their palladium complexes in 2012 and 2013 concerning the encapsulation of these complexes in a polymeric matrix and investigation of their catalytic activity in aqueous Suzuki–Miyaura cross-coupling reactions [181,182]. An example of synthesis of a palladium complex with pyridine functionalized NHC ligand is shown in Scheme 43. 

Three types of fully characterized palladium complexes, imidazolium based NHC with pyridine side chain, benzimidazolium based NHC with pyridine side chain and imidazolium NHC with pyrimidine side chain were incorporated into an amphiphilic media such as DSPE-PEG_2000_ polymer. The encapsulated complexes inside the hydrophobic cores of this medium showed high activity in cross-coupling reactions of a series of aryl halides with boronic acid in water. Using an identical pyridine functionalized NHC ligand, Xiang et al. also prepared a heterogeneous catalyst [182]. The major polymeric matrix was composed of *N*-(pyridin-2-ylmethyl)benzimidazolium chloride (NHC) and benzene that were cross linked by iron chloride as catalyst. Treatment with palladium ions caused its coordination to the functionalized NHC ligand, producing a heterogeneous catalyst that showed high activity in Suzuki–Miyaura crosscoupling of aryl halides with phenyl boronic acid in water. 

**Scheme 44:**
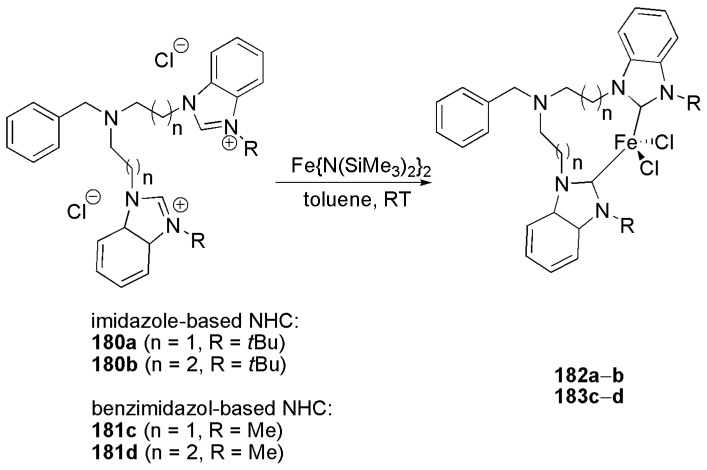
Bis-NHC ligands with flexible alkyl linkers [183].

To expand the scope of bidentate bis-NHC ligands and their iron complexes in catalytic reactions, Hazari et al. introduced novel bis-NHC ligands with flexible alkyl linkers, Scheme 44 [183]. 

Complexes **182**–**183** showed high activity in homocoupling of various aryl magnesium Grignard regents at 2 mol% catalyst loading within 30 min [183]. 

Singh et al. reported in 2013 a rare case of selenium containing bidentate NHC ligands [184]. This report served as the first example of palladium complexes stabilized with selenium containing NHC ligands. Moreover, by employing a chalcogenated ligand in designing palladium catalysts for cross-coupling reactions, a better knowledge of the formation of palladium nanoparticles, if any, and the mechanism of their formation could be achieved.

**Scheme 45:**
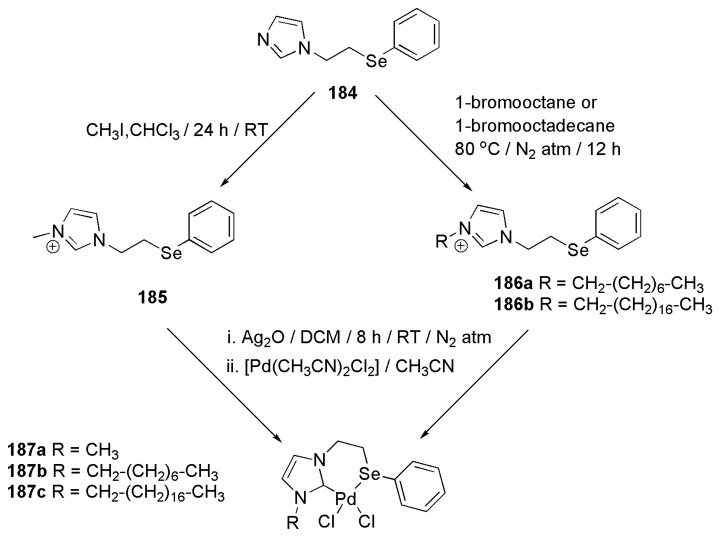
Synthesis of selenium containing NHC ligand [184].

N-alkylation of an imidazole and subsequent palladation via silver oxide route is shown in Scheme 45. The palladium complexes **187a**–**c** were succesfully employed in Suzuki–Miyaura cross-coupling reactions of a series of aryl aldehydes and phenyl boronic acids. Interestingly, a rapid formation of palladium nanoparticles at the beginning of catalytic reactions disclosed the role of selenium atom in the side chain and the length of R side chain in the overall activity of these catalysts. 

Bidentate methylene-bridged NHC ligands continue to experience structural modification to improve the stability of resulting metal complexes or their catalyst recoverability. While earlier changes in these ligands mostly focused on the wingtip (change of substituents on heterocycle nitrogen) [185,186], some groups focused on the backbone modifications [187]. Alternatively, bridging methylene group could also be tuned. In this regard, Kuhn et al. introduced a novel approach in 2014 [188]. 

**Scheme 46:**
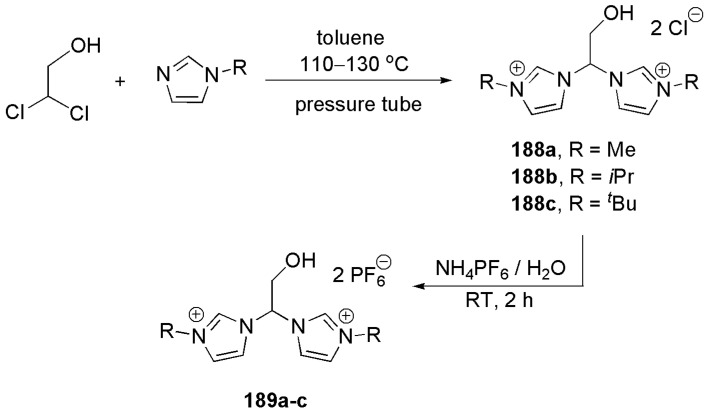
Novel bidentate methylene-bridged NHC ligands [188].

**Figure 21:**
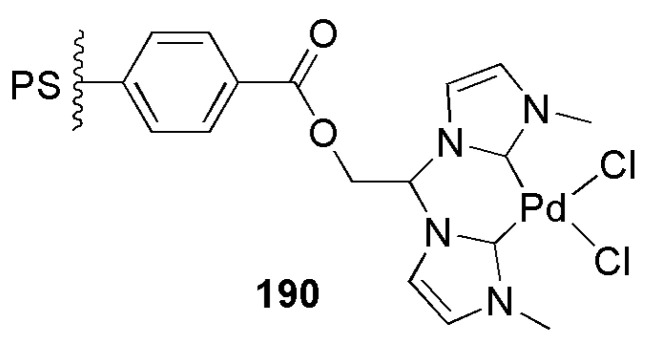
A palladium complex with bis-NHC ligand stabilized on polystyrene [188].

As shown in Scheme 46, hydroxyl functionalized bis-imidazolium salts were synthesized in a single step by the nucleophilic substitution reaction of dichloroethanol. More solubility was achieved with an anion exchange reaction with hexafluorophosphate. Out of two approaches, metalation and immobilization or opposite approach, immobilization and metalation, the latter was shown to be more effective in this case. Therefore, reactions with benzoyl chloride-functionalized polystyrene and subsequent metalation with palladium chloride provided complex **190**, Figure 21, which showed high activity in Suzuki–Miyaura coupling reaction [188].

In the same year, Yagyu et al. reported catalytic Heck cross-coupling application of palladium complexes supported with wingtip modified methylene bridged bis-NHC ligands [189]. 

In an interesting paper, Ruzicka et al. disclosed the crucial role played by the presence of donor sites in an NHC ligand bearing a heteroatom-containing arm [190]. 

**Scheme 47:**
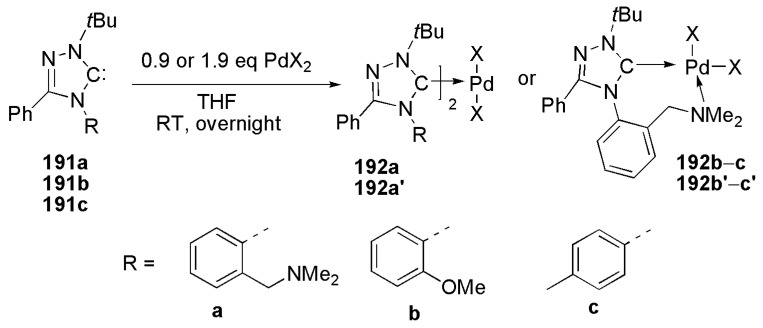
Formation of Pd Complexes with free carbenes [190]. X = Cl for **192a**–**c**, OAc for **192a′**–**c′**.

As shown in Scheme 47, the coordination mode of free carbene, generated by the deprotonation of the parent triazolium salt, is dependent upon the type of side arm heteroatom. With an amino group, chelating NHC ligand forms a rare seven-membered ring (**192a** or **192a**’), while the presence of 4-methyl phenyl or methoxy-phenyl substituents on triazole based NHC ligand, caused it to act as a monodentate ligand. chelating NHC ligands (**4a** or **4a**’) showed superior activity compared to bis-NHC palladium complexes in catalytic Suzuki–Miyaura reactions. This observation revealed that functionalized triazole-based NHC ligands have significant impact in their catalytic performance. 

**Figure 22:**
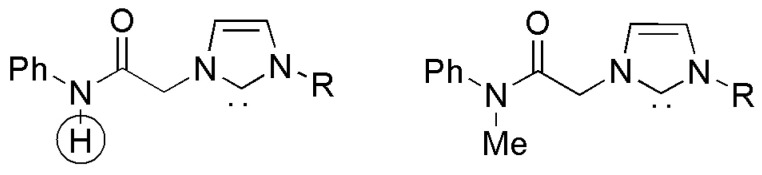
Side chain influence in an NHC ligand on catalytic activity of palladium complex. [191].

Structurally identical to their earlier report in 2010, palladium(0) complexes supported with acetamide functionalized NHC ligands were reported in 2015 by Lee et al. [191]. The catalytic activity of novel palladium(0) complexes bearing *N*-methyl-*N*-phenylacetamide side chain (right, Figure 22) in Mizoroki–Heck type reactions was shown to be inferior than that of N-phenylacetamide (left, Figure 22). The influence of this small change was attributed to deprotonation of N-H moiety and generation of electron rich species during catalytic reactions.

**Figure 23:**
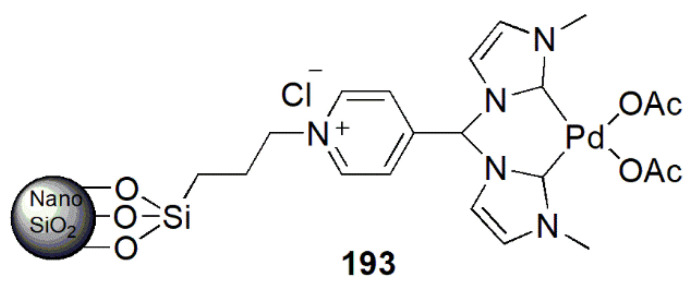
**A bis-NHC** palladium complex supported on nanomagnetic particles [192].

An alternative approach to functionalize bridging group in a bis-NHC ligand and its palladium complex was reported by Moghadam et al. in 2016 [192]. The general structure of this novel catalyst, **193** in Figure 23, contains a pyridine in the bridging methylene, allowing its loading on a magnetic nanoparticle for better recyclability in catalytic coupling reactions. This catalytic system showed high activity in the Suzuki–Miyaura coupling of phenylboronic acid with a series of aryl halides.

**Scheme 48:**
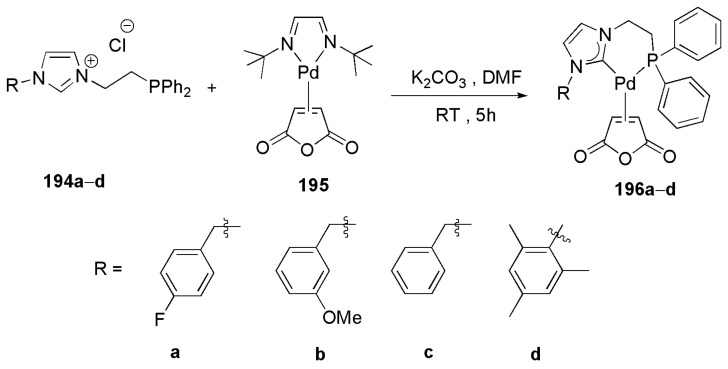
Chelation of bidentate NHC-PR_2_ ligands onto Pd(0) [193].

A bidentate NHC-PPh_2_ ligand has been utilized for the preparation of palladium(0) complexes to be used in Heck reactions [193]. The imidazolium salts **194** were prepared starting from N-substituted imidazoles which were first heated at 90 °C for 24 h in 1,2-dichloroethane to afford (β-chloroethyl)imidazolium chlorides. The latter compounds were then transformed into **194** by reaction with KPPh_2_ [193]. Reaction of deprotonated **194** with the palladium(0) precursor **195** in DMF yielded the desired Pd(0)-NHC-PR_2_ complexes **196** (Scheme 48).

Studies on the catalytic activities of Pd(0)-NHC-PR_2_ complexes **196a**–**d** in Mizoroki–Heck coupling reactions revealed their superiority compared to the corresponding palladium(0) complexes with diphosphine ligands.

**Scheme 49:**
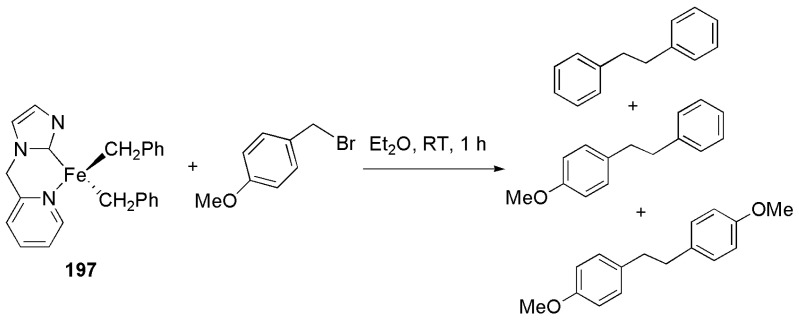
Novel iron(II)-NHC for Kumada-type coupling reactions [194].

An interesting Kumada-type coupling reactions was studied using the iron(II)-NHC complex **197**, which was prepared by reaction of NHC-FeBr_2_ with benzyl magnesium bromide [194]. Complex **197**, reluctant to benzyl ligand dissociation upon reaction with a strong σ donor and π acceptor such as carbon monoxide, was shown to be effective in the Kumada coupling of substituted benzyl bromides. As shown in Scheme 49, treatment of the iron-NHC complex with benzyl bromide yielded a mixture of coupling products. This is perhaps a rare example of a metal complex supplying part of reaction substrate and catalyzing coupling reaction, simultaneously. 

**Figure 24:**
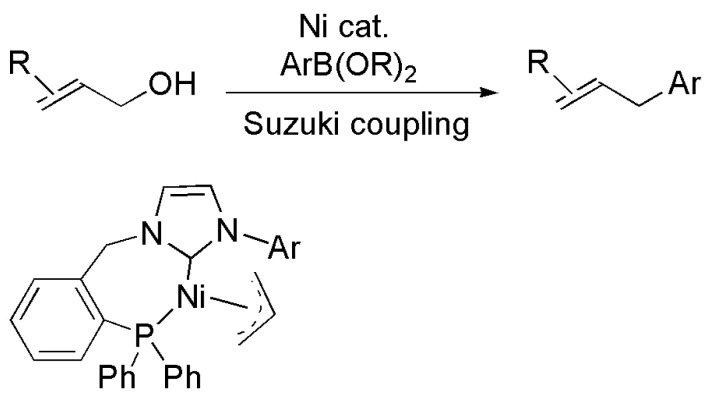
Nickel-based NHC ligand for Suzuki coupling [195].

Zhang et al. reported an innovation in nickel catalyzed Suzuki–Miyaura reactions of unprotected alcohol electrophiles in 2016 by the introduction of highly active nickel complexes supported with bidentate NHC-phosphorous donor ligands. The general structure of the catalyst is shown in Figure 24 [195]. By employing bidentate NHC-phosphine ligand, the coupling of a variety of unprotected alcohols with boronic esters were carried out with high efficiency. Again, the bidentate nature of the ligand, providing more stability to the nickel catalyst, is considered a key advantage during this process. 

**Scheme 50:**
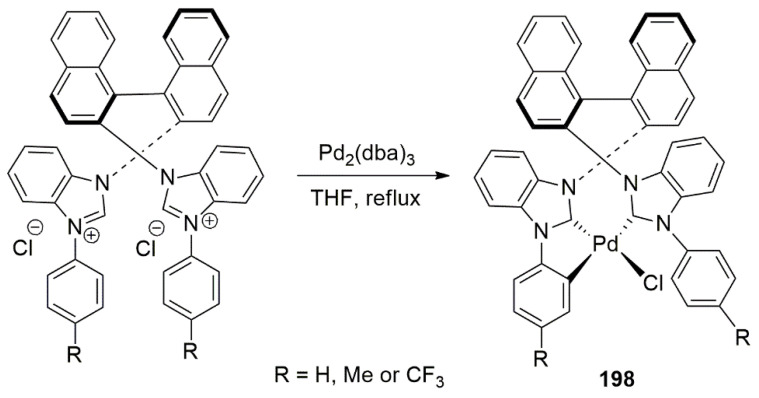
Chiral NHC ligands for catalytic asymmetric aryl–aryl coupling reactions [196]. dba: dibenzylideneacetone.

The use of chiral NHC ligands for catalytic asymmetric aryl-aryl coupling reactions have gained some attention during past decade especially with the development of efficient bis-NHC ligands with chiral linkers. In this regard, asymmetric coupling reactions using chiral palladium complexes with NHC ligands are attracting a lot of attention. A recent example in this regard includes the work of Zhang et al. in 2017, when they introduced novel N,N’-bisaryl substituted bis-NHC ligands and their palladium complexes **198** (Scheme 50) for efficient catalysis of asymmetric aryl–aryl cross-couplings [196]. This research showed the vital role played by chiral linkers in this type of enantiocatalysis, although the details are not yet fully understood. 

**Figure 25:**
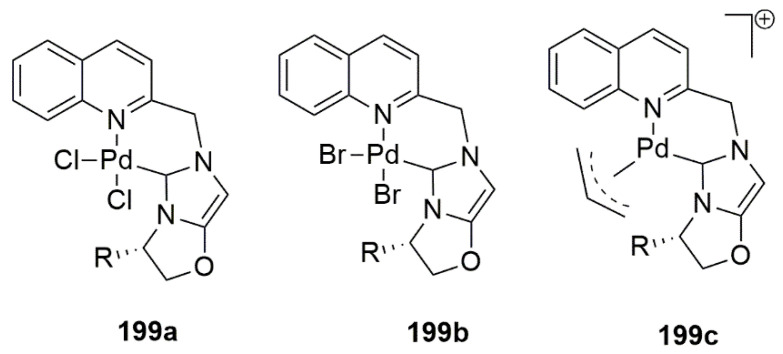
Pd complexes bearing chiral bicyclic imidazoles [197].

Michael et al. developed a related ligand system in 2017 where they have intended to control relative orientation of chiral substituent in a key step for catalytic enantioselective reactions. The complexes designed for this purpose, bearing chiral bicyclic imidazoles, are shown in Figure 25. A polycyclic NHC with tethered nitrogen donors enforces a constrained geometry around palladium complex via restricted rotation of the chiral moiety and nitrogen on fused ring [197]. A flexible tethered group of NHC ligand also provides a large bite angle that confers high stability to the complex.

**Scheme 51:**
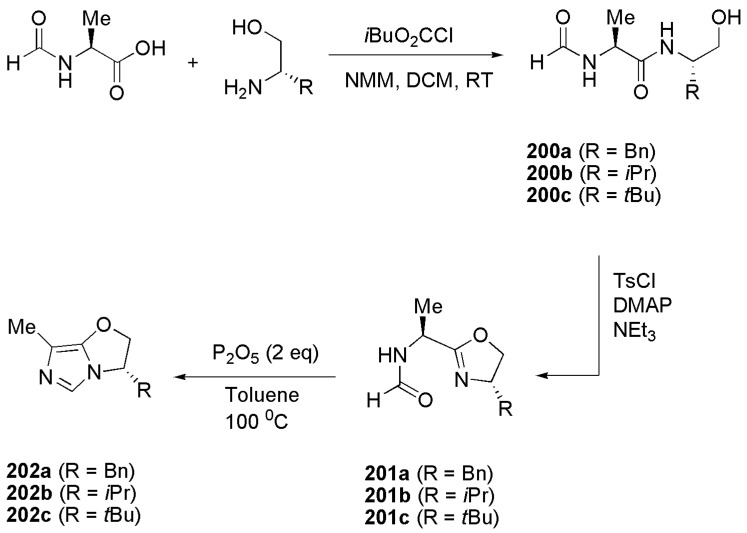
Synthetic route to oxazolines **202a**–**c** [197].

The oxazolines **201a**–**c** necessary for the synthesis of the chiral bicyclic imidazoles were prepared as shown in Scheme 51. In the first step, reaction of amino alcohols with N-formylalanine yields diamides **200a**–**c**. Tosyl chloride mediated cyclization of diamides to oxazoline and subsequent dehydration with P_2_O_5_ affords bicyclic structures **202a**–**c**. 

**Figure 26:**
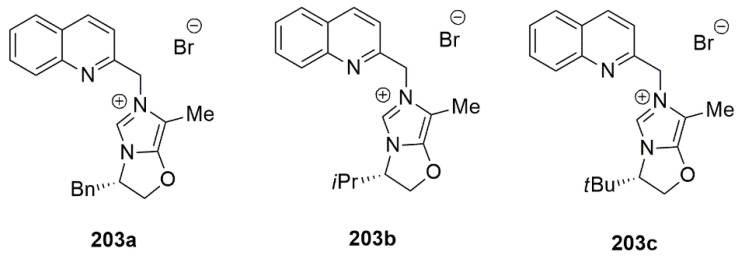
Synthetic route to bicyclic imidazoles [197].

Alkyl imidazoles **203a**–**c** (Figure 26) were achieved by reacting **202a**–**c** with 2-(bromomethyl)quinoline. 

The reactivity of palladium complexes **199a**–**c** was investigated in allylic amination and alkylation of cyclohexenyl methyl carbonate, and no activity was observed. These complexes were also inactive in the Hartwig–Buchwald coupling reactions which is surprising considering reported catalytic activities of previously reported NHC palladium complexes. 

Catalyst recovery and metal content removal of an organic product is a challenge for chemists particularly in large industrial-scale reactions catalyzed by homogeneous metal catalysts. There are different strategies for catalyst recovery and despite progresses made in this regard, each strategy has its own drawbacks. Organic solvent nanofiltration (OSN), because of its simplicity, has been used as an attractive technology for the recovery of soluble organometallic catalysts. One trick for optimal use of this technology was reported in 2018 by Jesus et al. that have developed high molecular weight catalysts via manipulation of ligand structures used in the construction of organometallic catalyst [198]. 

**Scheme 52:**
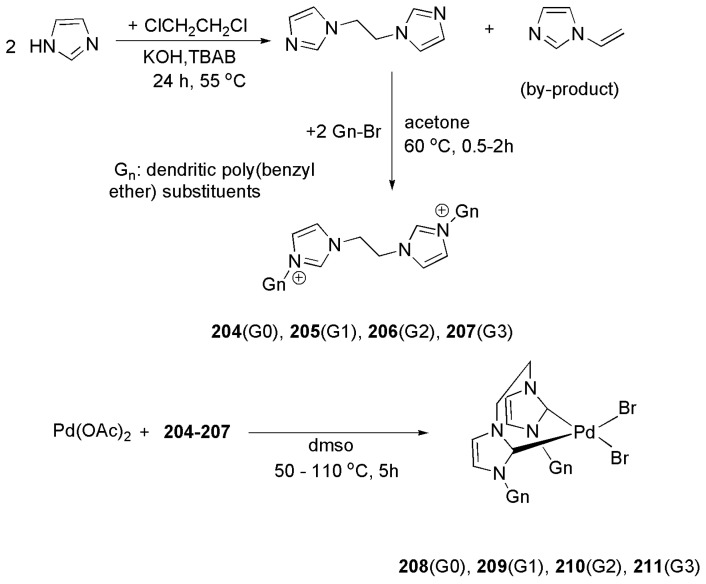
Bis-NHC ligands and their coordination to palladium [198].

**Figure 27:**
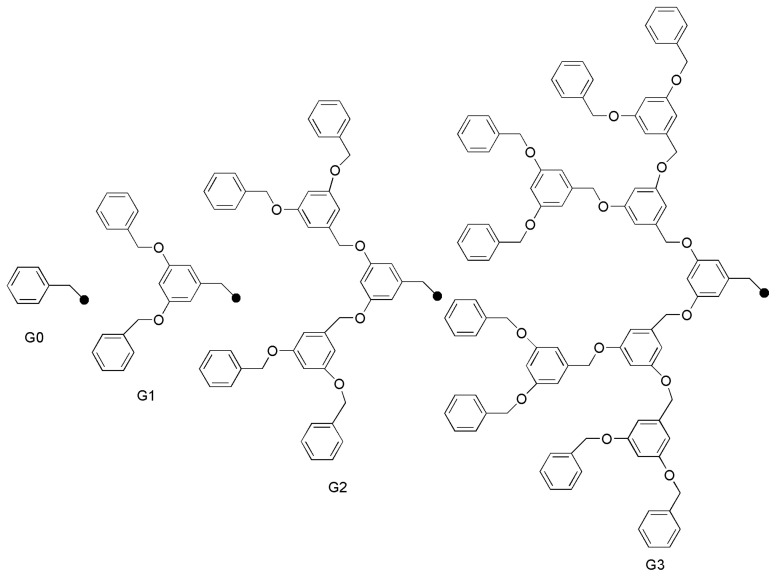
Structures of dendrons used for the synthesis of bulky bis(imidazolium) salts [198].

The design principle for such catalysts is shown in Scheme 52. In the first step, two equivalents of imidazole react with 1,2-dichloroethane. Then, reaction of ethylene bridged bis(imidazole) with two equivalents of the dendrons, depicted in Figure 27, yield bulky bis(imidazolium) salts **204**–**207**. 

Based on a procedure developed by Herrmann et al., bis(carbene) palladium complexes **208**–**211** were prepared by direct metalation of imidazolium bromide salts using palladium acetate (Scheme 52). 

**Figure 28:**
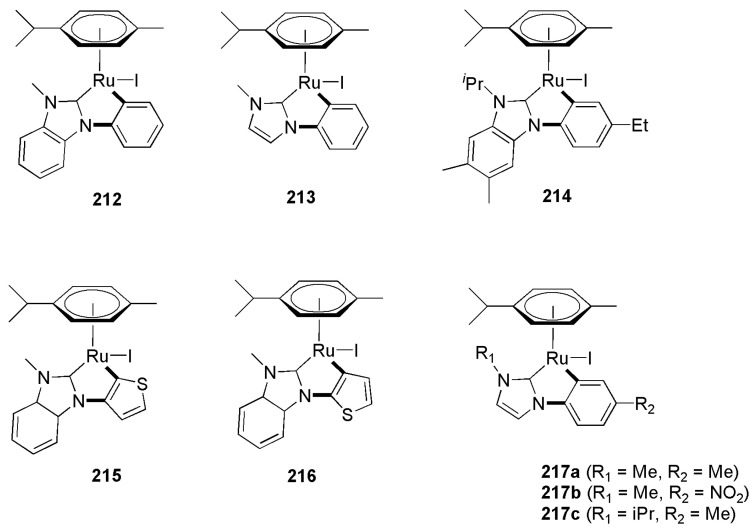
Bidentate C^NHC^-C-based ligands introduced by Verpoort et al. [199].

A series of highly active Ru-based catalysts with NHC-carbon donor ligands in dehydrogenative coupling reactions was reported by Verpoort et al. in 2019 [199]. This group, earlier in 2019, utilizing identical catalysts, reported efficent dehydrogenative coupling of alcohols and amines to form amidates [200]. Although reports regarding these types of coupling reactions are prevalent, most of the known efficient catalysts utilize mono-dentate NHC ligands or phosphine-based ligands. However, the use of bidentate C^NHC^-C based ligands and their ruthenium complexes as catalysts, Figure 28, showed higher activity at low catalyst loadings (as low as 250 ppm in alcohol and hydroxide coupling reactions) compared with that of previously reported cases [199]. 

Most recent reports regarding functionalized NHC ligands, related palladium complexes and their application in C-C coupling reaction have been published by Engle et al. and Deydier et al. [201,202]. 

### 2.4. Ethylene Transformation

The introduction of *ortho*-aryloxide-NHC ligands by Grubbs et al. in early 21st century provided an alternative ligand system to the salicylaldimine framework [203]. The investigation of nonmetallocene based olefin polymerization systems took a new turn by the introduction of catalysts based on early transition metals bearing salicylaldimine ligands [63]. N-heterocyclic carbene ligand with *ortho*-hydroxy aryl tether are analogous to salicylaldimine ligands and were used extensively in the olefin polymerization reaction. Earlier reports regarding the use of titanium and zirconium complexes with bidentate NHC-phenoxide ligands as polymerization catalysts appeared in 2011 [63]. 

**Scheme 53:**
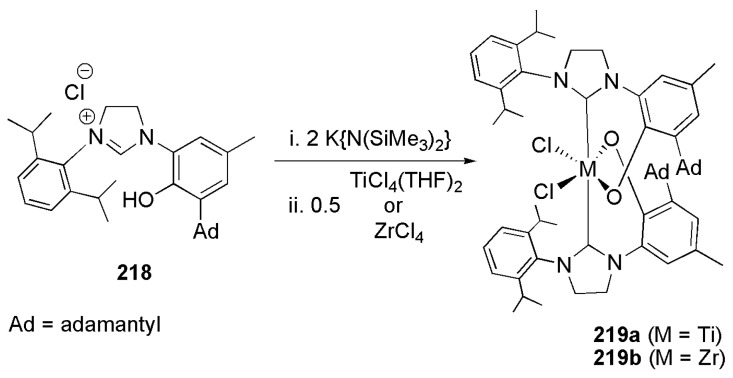
Ti and Zr complexes with *ortho*-aryloxide-NHC ligand for olefin polymerization reactions [63].

As shown in Scheme 53, double deprotonation of ligand precursor **218** with a strong base and subsequent reaction with TiCl_4_(THF)_2_ or ZrCl_4_ yielded the corresponding titanium and zirconium NHC complexes. These complexes were active catalysts in the polymerization of ethylene. The copolymerization of ethylene with 1-octene and norbornene was also caried out successfully with these catalytic systems. 

Using a similar approach and to find an alternative and more active catalysts for the polymerization of styrene, Albrecht et al. in 2012 reported successful application of bidentate NHC-pyridine ligands and their palladium complexes in the styrene polymerization [204]. While reports of palladium with a-diamine ligands (Brookhart’s catalyst) or Drent’s phosphinosulfonate based ligands were successfully applied in terminal alkene polymerization, the development of bidentate NHC-N donor ligands and their palladium complexes provided an opportunity to compensate for some of the drawbacks of the previous catalytic systems.

**Scheme 54:**
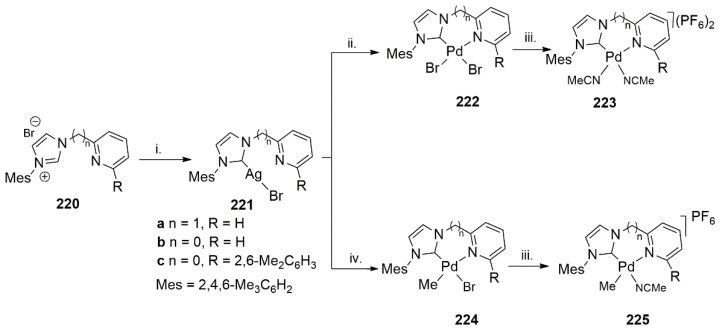
Palladium complexes with NHC-N donor ligands for styrene polymerization [204]. Reagents and conditions: (i) Ag_2_CO_3_, CH_2_Cl_2_, reflux; (ii) [PdBr_2_(cod)], CH_2_Cl_2_; (iii) AgPF_6_, MeCN; (iv) [PdMeBr(cod)], CH_2_Cl_2_ or MeCN.

Two different procedures, shown in Scheme 54, were utilized to synthesize palladium complexes with NHC-N donor ligands with different steric and electronic properties.

Out of multiple metal complexes shown in Scheme 54, **225b** and **225c** were shown to be highly active in the polymerization of ethylene and styrene with opposite effects [204].

**Scheme 55:**
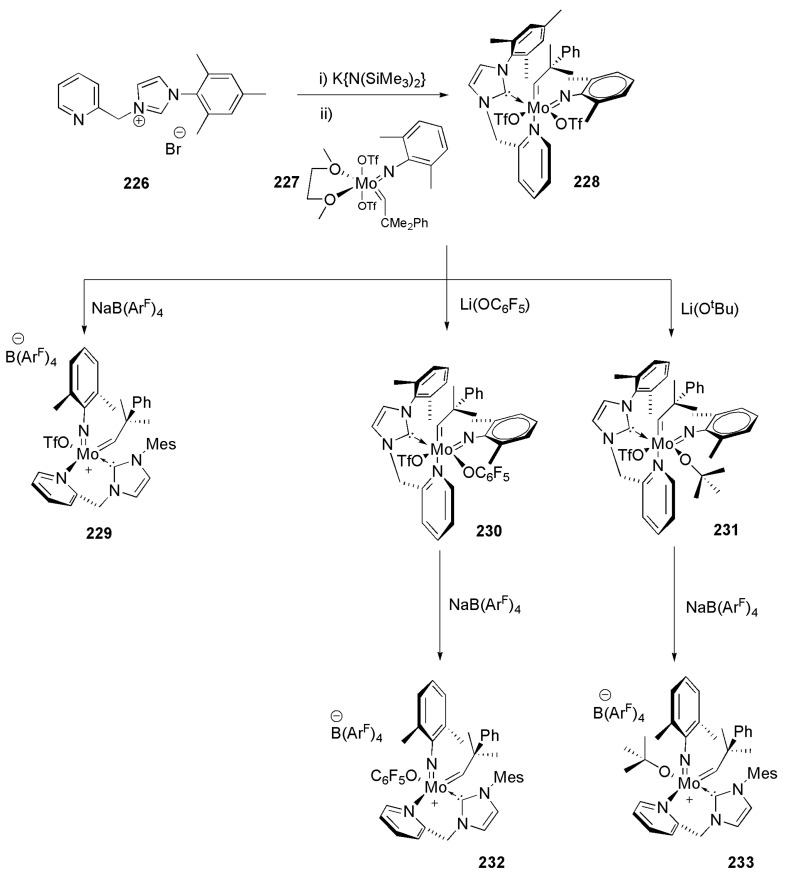
Synthesis and derivatization reactions of molybdenum alkylidene complex **228** [205].

Group six alkylidene complexes, in particular molybdenum and tungsten alkylidenes were investigated largely for their role as initiators in catalytic olefin metathesis reactions. To investigate whether the presence of a chelating NHC-pyridine ligand could enforce extra stability to molybdenum imido alkylidene complexes, Buchmeiser et al. explored the coordination ability and the reactivity of the bidentate NHC-pyridine ligand **226** (Scheme 55) [205]. The in situ generated NHC ligand bearing a tethered pyridine displaces dimethoxyethane (DME) in molybdenum precursor **227** yielding the six-coordinate complex **228**, which has two triflates and an alkylidene in the coordination sphere. In the single crystal X-ray analysis of **228**, pyridine from bidentate ligand is shown to be situated in *trans* position to the alkylidene ligand, providing extra evidence of the way, *trans* to alkylidene, the olefin substrate approaches the metal center in metathesis reactions [205]. Substitution of a triflate group by nucleophiles such as alkoxides provided a way to derivatize **228**, with formation of products that can have huge impact on the structure and reactivity of the original metal complex. Electron withdrawing pentafluorophenoxide and electron donating *tert*-butoxide were utilized for this purpose.

Among the cationic Mo(IV) complexes **229**, **232**, and **233**, a higher activity in the ring closing metathesis (RCM) of 1,7-octadiene at 80 °C was shown by species **229**.

Using analogous molybdenum imido alkylidene N-heterocyclic carbene complexes, this group also reported successful homo-metathesis of 1-octene and 1-nonene [206].

**Figure 29:**
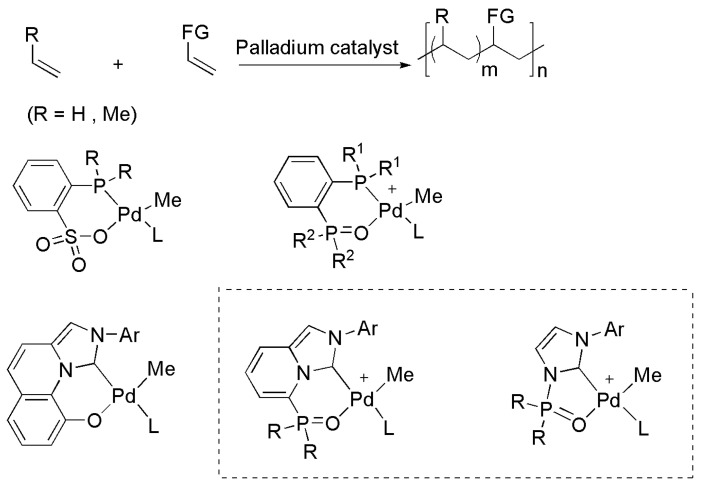
Design strategy for NHC-oxygen ligand [208].

During the past two decades, group 10 metal complexes bearing unsymmetrical bidentate ligands have played an important role in the catalytic copolymerization of polar olefin substrates [207]. As shown in Figure 29, earlier examples of bidentate ligands utilized for this purpose included soft sigma donor phosphines together with hard sigma donor ligands such as sulfonates [208]. A key point to the success of these catalytic systems in this particular reaction is to design a ligand system with enough chelation power to not only thermally stabilize metal ions but also provide a rigid environment around metal center so that some decomposition pathways for the metal complex, such as reductive elimination, is blocked. This strategy was implemented by Nozaki et al. and their ligand design concept involved tethering a phosphine oxide, through a rigid backbone, to an N-heterocyclic carbene to access a bidentate ligand suitable for group 10 metal ions [208]. 

**Scheme 56:**
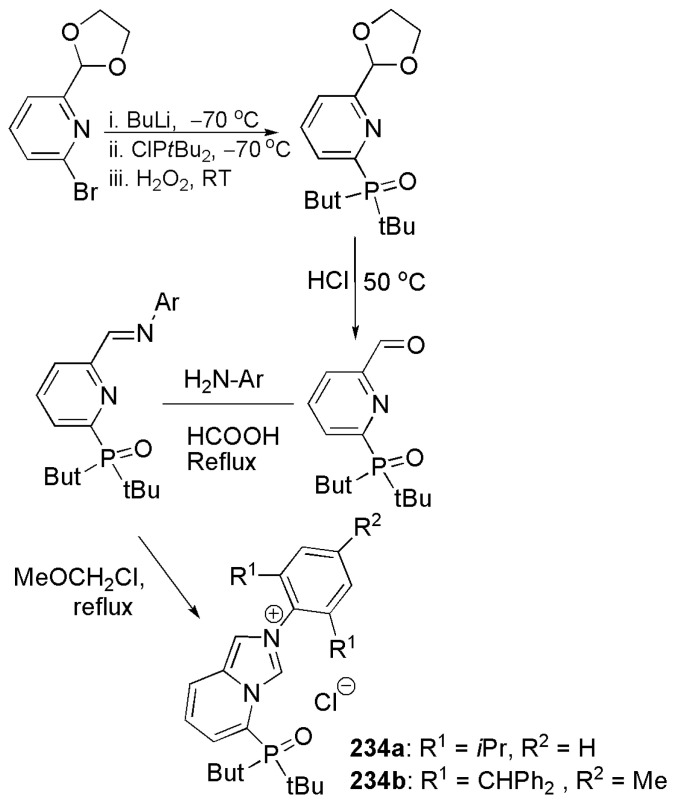
Synthetic strategy for bidentate NHC-PR_2_(O) ligands [208].

As depicted in Scheme 56, *ortho*-lithiation of 2-bromo-6-(1,3-dioxolan-2-yl)pyridine and subsequent nucleophilic attack of carbanion on di-tert-butylphosphinic chloride introduces di-tert-butylphosphine oxide group. Subsequent deprotonation, Schiff base condensation and cyclization with methoxymethyl chloride yielded the desired rigid imidazolium salts **234a** and **234b** with a side phosphine oxide group as a weak sigma donor site [208].

To obtain palladacyclic complexes, ligand precursors **234a** and **234b** were deprotonated with bis(trimethylsilyl)amide, then reacted with silver(I) salts. Transmetalation with palladium salt furnished the desired product **235**.

**Scheme 57:**
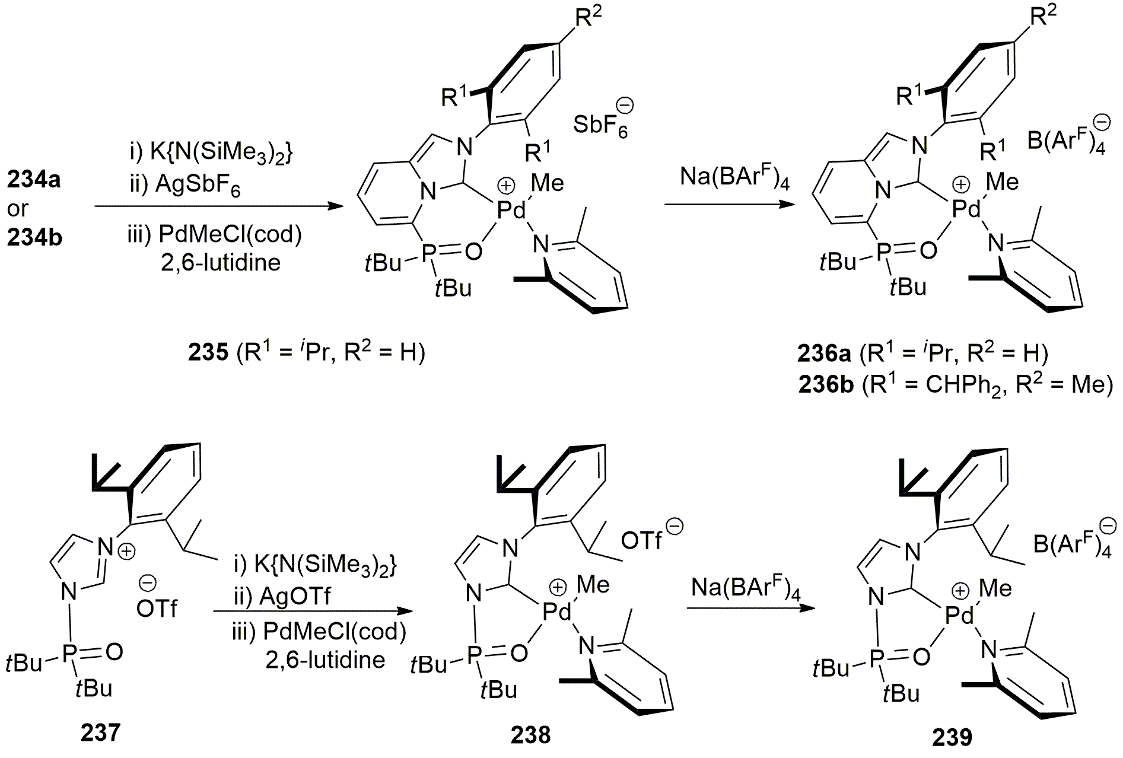
Palladium complexes bearing NHC-PR_2_(O) ligands [208].

Complexes **235**, **236a**, **236b,** and **239** in Scheme 57 were utilized as catalysts for ethylene polymerization and ethylene/polar monomer copolymerization. Complex **235**, with a six-membered chelate ring, exhibited a good catalytic activity for ethylene polymerization while complex **239**, with a five membered chelate ring, exhibited low catalytic activity.

**Scheme 58:**
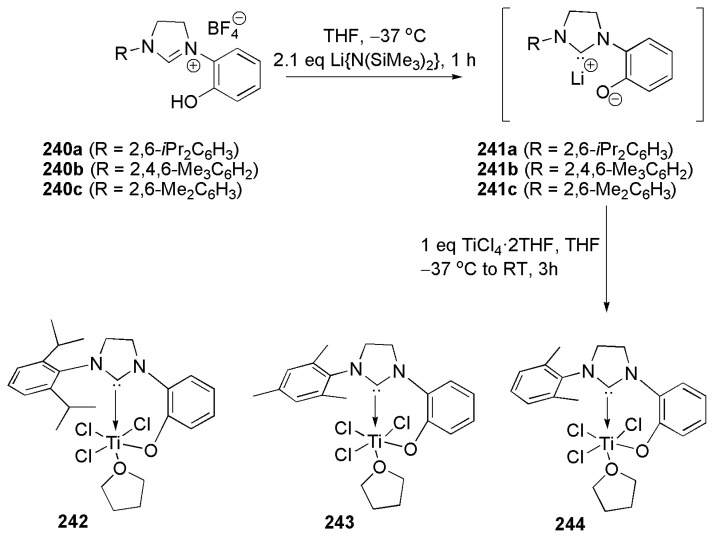
Synthesis of titanium(IV) complexes [210].

Titanium complexes **242**–**244** (Scheme 58) were prepared according to the procedure introduced by Grubbs et al. in 2004 [209]. Double deprotonation of imidazoline salts **240a**–**c** with Li{N(SiMe_3_)_2_} and reaction with TiCl_4_ led to the isolation of red-colored, six-coordinate titanium complexes [210].

**Scheme 59:**
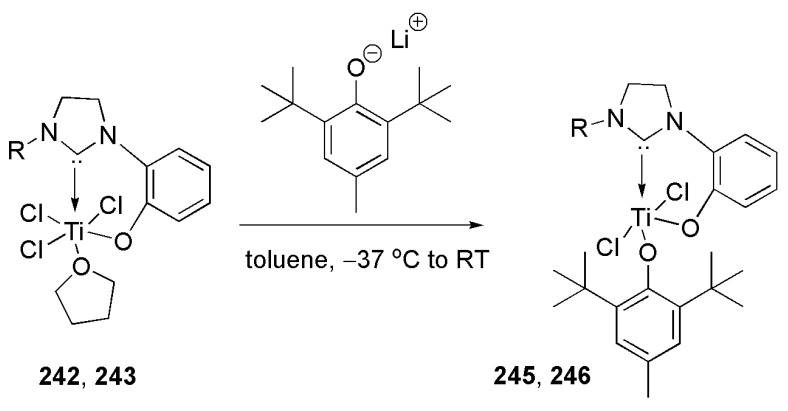
Synthesis of mixed ligand Ti(IV) complex [210]. **242**, **245**: R = 2,6-*i*Pr_2_C_6_H_3_; **243**, **246**: R = 2,4,6-Me_3_C_6_H_2_.

Bulky substituents on imine nitrogen of “post-metallocene catalysts” bearing salicylaldimine ligands play a vital role on the catalytic activity of these systems. To achieve a more soluble catalysts and to improve their catalytic activities, the chloro ligand in **242** and **243** was substituted with a bulky aryloxo ligand via simple reaction with lithiated salt of aryloxo ligand, leading to complexes **245** and **246** (Scheme 59). 

In the homopolymerization reaction of ethylene and its copolymerization with cyclic olefins, catalyst **245**, with a bulky aryloxo ligand *trans* to NHC ligand, afforded high-molecular weight polymers and copolymers, by suppressing the pathway for β-hydride elimination [210].

Employing an identical bidentate and unsymmetrical *o*-hydroxyaryl-substituted N-heterocyclic carbene ligands, bis-ligated titanium(IV) metal complexes, Le Roux et al. reported moderate catalytic activities in ethylene polymerization [211].

**Scheme 60:**
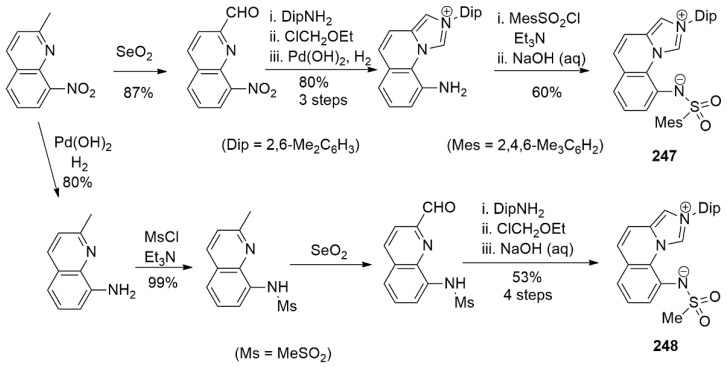
Synthesis of sulfonamide pre-ligands [212].

Incorporation of strong and weak σ-donors in an unsymmetrical bidentate ligand has led to substantial increase in the catalytic activity of certain metal ions such as palladium in the copolymerization of a polar monomer and olefins. In this regard, the development of NHC ligands incorporating a weak σ-donor such as sulfonamide was investigated. NHC moiety in these ligands, owing to its strong σ donation capacity, can replace phosphine or α-diimine ligands in older catalytic systems developed for these particular catalytic reactions. Besides, bidentate ligands based on NHC-sulfonamide donors provide sufficient structural integrity for the catalyst system, therefore increasing its performance during catalytic reactions. Nozaki et al. published an interesting report in this regard in 2018 where they describe a couple of novel bidentate NHC-sulfonamide ligands with rigid backbone (**247** and **248**, Scheme 60) to stabilize palladium ions in a square planar coordination environment [212]. The nature of alkyl sulfonyl substituent (Mes or Me) makes a meaningful difference in terms of reaction outcome and different synthetic routes were implemented to achieve imidazolium salts **247** and **248** in adequate yields. 

**Scheme 61:**
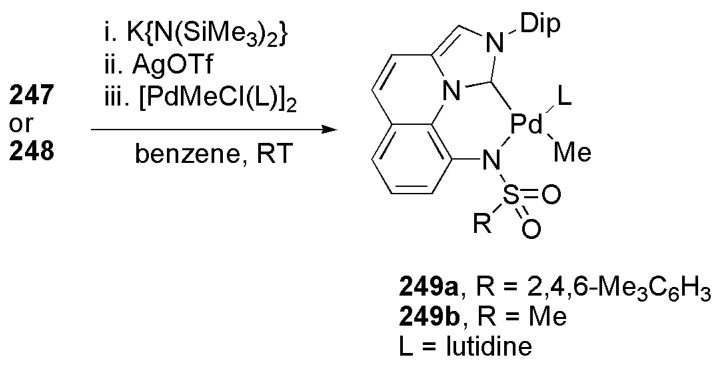
Synthesis of palladium complex with bidentate NHC ligand [212].

The general method involving silver-carbene intermediates was utilized to successfully achieve Pd(II) NHC-sulfonamide complexes **249a**–**b** (Scheme 61).

Palladium complex **249a** showed superior catalytic activity with respect to **249b** for the incorporation of polar substrates in ethylene polymers [212].

Fundamental research concerning the effect of spacers in the ether or amine functionalized NHC ligands was reported by Braunstein et al. in 2019. While in ether functionalized ditopic NHC ligands, where an ether group is attached to the nitrogen atom of the heterocycle, the oxygen atom remains uncoordinated [213]. On the other hand, in alcoholate functionalized NHC ligands with two or three atom carbon spacers, this oxygen atom is coordinated and the whole ligand shows a chelating behavior. 

Since the number of spacer carbon atoms plays an important role in chelation of functionalized NHC ligand, a series of NHC ligands with etherate groups were prepared and their coordination to silver as well as nickel ions was investigated. It was concluded that in this case, despite overall similarities between ether and alcohol functional groups as side chain in NHC ligands, the oxygen atom in the latter one remains uncoordinated. On the other hand, the nitrogen atom in amine functionalized NHC ligand causes a k^2^ NHC-N coordination mode in the ligand system. This coordination mode in the nickel complexes of ligands with k^2^ NHC-N coordination mode, caused an improvement in their catalytic ethylene oligomerization, a property that was not observed with similar ligands containing dangling etherate groups [213]. 

### 2.5. Other Applications of Bidentate NHC Ligands 

Research areas where metal mediated organic molecule transformations were conducted using bidentate NHC ligands to a lesser degree include reduction of carbonyl compounds using hydrosilylation process [209,214,215,216,217,218,219], isomerization of allylic alcohols [220], cross metathesis [221], arene borylation [222,223], amination [224], reduction of sulfoxides [225], alcohol oxidation [132,226,227], CO_2_ reduction [228,229,230,231], norbornene polymerization [232,233,234], alkylation of amines [235], C-H borylation [236], CO_2_ activation [237], hydroamination [238], functionalization of propane [239,240], ylide cycloaddition [74], three component coupling [241], hydroxylation of benzene [242], hydroaminoethlyation [243], water oxidation [244], amine alkylation [245], homopolymerization of ethylene [246], destruction of chemical warfare [247], Friedel-Crafts alkylation [248], C–H arylation [249], C–H oxidation [250], SABRE catalyst [251], and alcohol amidation reactions [211]. Some examples from these applications are discussed in the following.

**Scheme 62:**
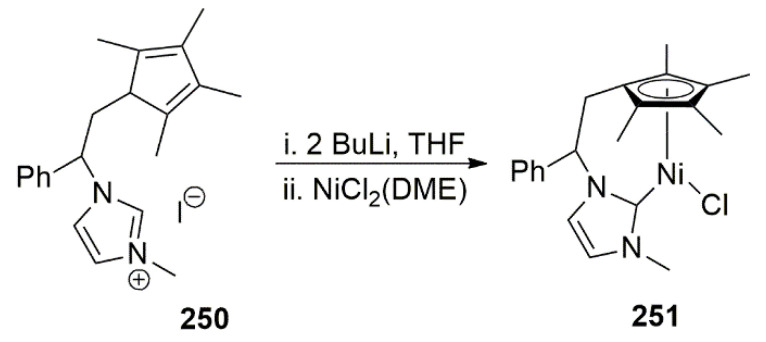
Synthesis of a nickel complex bearing bidentate NHC-Cp ligand [225] DME = dimethoxyethane.

The well-defined nickel complex **251** bearing a NHC ligand with an anionic cyclopentadienyl tether was reported by Royo et al. in 2012 (Scheme 62) [225]. Complex **251** was utilized in the hydrosilylation of aldehydes and ketones.

**Scheme 63:**
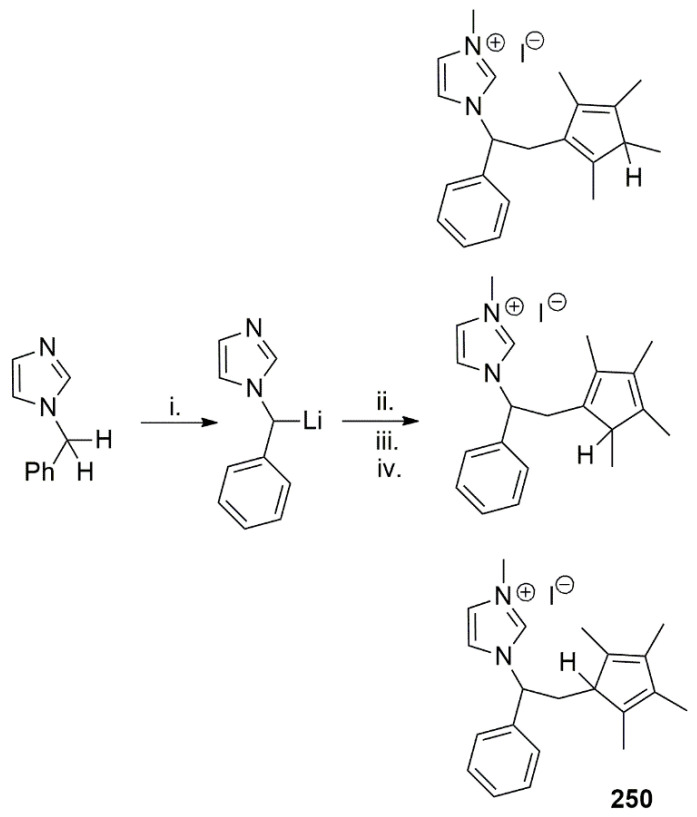
Synthesis of proligand **250** [113]. Reagents and conditions: (i) *n*BuLi; (ii) tetramethylfulvene; (iii) MeOH; (iv) MeI.

The same group earlier described the synthesis of a bidentate pro-ligand to stabilize nickel ion. The imidazolium pro-ligand **250** was obtained by lithiation of the methylene group of benzylimidazole with *n*-BuLi and subsequent reaction with tetramethylfulvene in MeOH. A mixture of tautomers was obtained upon reaction with MeI in acetone (Scheme 63) [113].

**Scheme 64:**
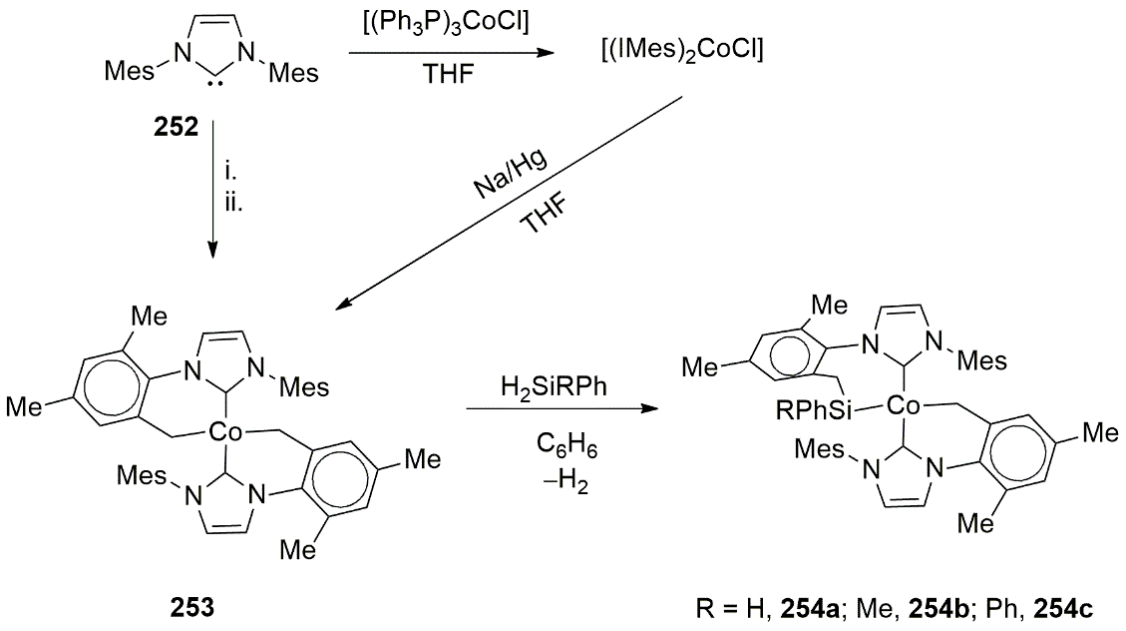
Synthesis of Co(II) complexes bearing silyl-functionalized NHC [214]. Reagent and conditions: (i) CoCl_2_, THF; (ii) Na/Hg.

To prepare a bidentate ligand of the type NHC-silyl to support cobalt(II) ions, Deng et al. carried out cobalt-mediated silylation of benzylic C–H bonds [214]. Sodium amalgam reduction of a methyl group of the IMes ligand **252** resulted in the square planar cyclometalated cobalt(II) complex **253** (Scheme 64). This single step route requires cobalt(II) chloride as cobalt ions source. The same product **253** could be obtained also in two steps by reaction of **252** with (PPh_3_)_3_CoCl followed by Na/Hg reduction. The addition of H_2_SiRPh, (R = H, Me, Ph) to a benzene solution of **253** led to the formation of NHC-silyl chelated cobalt complexes **254a-c** (Scheme 64), which were utilized as catalyst for the hydrosilylation of 1-octene [214]. 

**Scheme 65:**
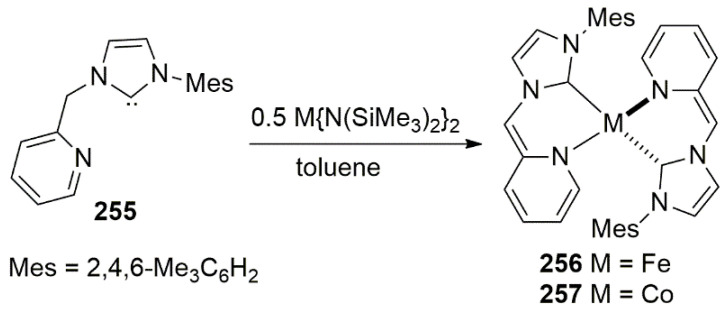
Iron and cobalt coordination of a free NHC-*N*-picolyl ligand leading to tetracoordinated complexes [215].

*N*-picolyl NHC ligand **255** and its complexation onto iron(II) or cobalt(II) to furnish low coordinate complexes **256** and **257** (Scheme 65) was reported by Song et al. [215]. Free carbene ligand was prepared by deprotonation of the imidazolium salt parent in ether at room temperature. The chelating or non-chelating behavior of **255** towards metal salt depends on the ratio of reagents. The reaction with a ligand to metal ratio of 2:1 provides a low coordinate iron and cobalt complex in which tethered picolyl loses aromaticity upon chelation (Scheme 65). 

**Scheme 66:**
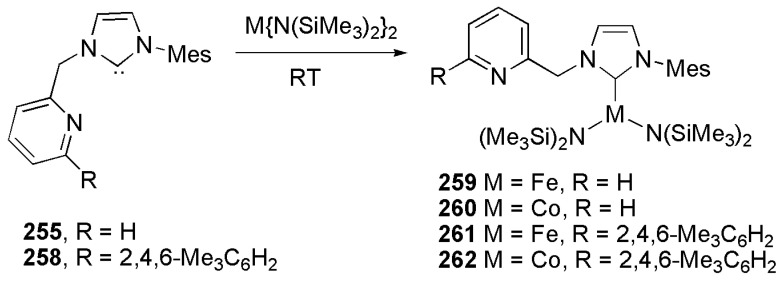
Iron and cobalt coordination of free *N*-picolyl NHC ligands leading to tricoordinated complexes [215].

As similar reaction, but with equimolar amounts of *N*-picolyl NHC ligand and metal, leads to three coordinate iron and cobalt complexes with *N*-picolyl NHC ligand coordinating in a monodentate mode (Scheme 66). 

**Scheme 67:**
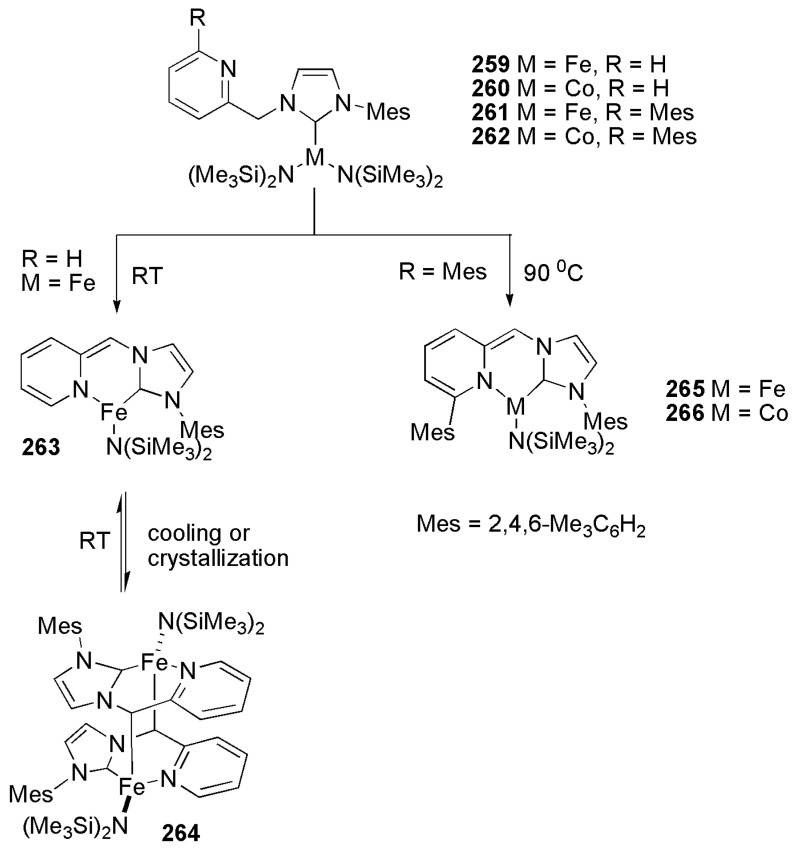
Reactivity of NHC-*N*-picolyl ligands with iron and cobalt salts [215].

The effects of reaction time and temperature on tricoordinate iron and cobalt complexes are shown in Scheme 67. The kinetically stable tricoordinate complex, with no substituents on tethered picolyl group, turned into stable tricoordinate complexes in which picolyl is also chelated after few hours. The low temperature crystallization of the chelate iron complex leads to the dimeric structure **264**. For tethered picolyl ligands with sterically demanding mesityl substituents, chelation only occurs at high temperature [215]. 

The catalytic activity of cobalt and iron complexes with chelate bidentate NHC ligands were screened in the hydrosilylation of ketones. The results revealed the iron complex was the most effective at a low catalyst loading of 0.05 mol% at ambient temperature [215]. 

In another research, the synthesis and characterization of iron complexes with NHC-Cp ligands was reported in 2013 [216]. The iron complexes in combination with potassium *t*butoxide showed high activity in the reduction of ketones. 

**Figure 30:**
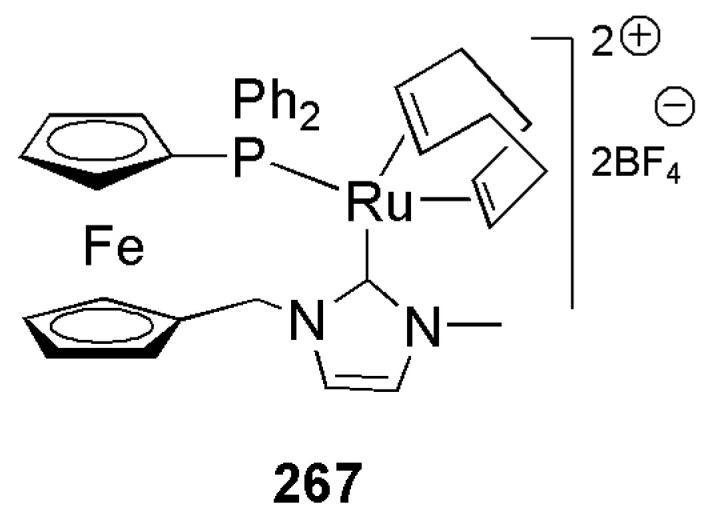
Ruthenium complex of redox-active ferrocenyl phosphine-NHC ligand [217].

Redox-active ferrocenyl phosphine-NHC ligand was employed by Labande et al. to prepare a Ru(III) complex which turned out to be active in the hydrosilylation of acetophenone derivatives [217]. The structure of this interesting ligand and its ruthenium complex **267** is shown in Figure 30. Previous reports about this ligand revealed its utility in a handful of fundamental research by several research groups [13,252]. 

**Scheme 68:**
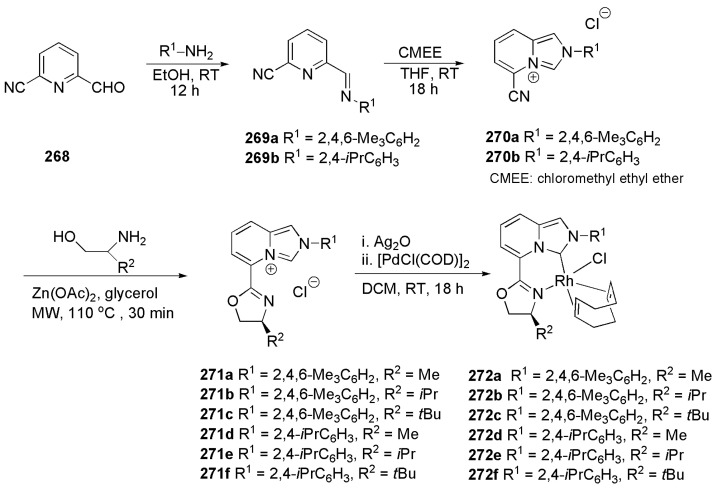
Synthesis of chiral salts and corresponding rhodium(I) complexes [218]. CMEE: chloromethyl ethyl ether.

A recent example of a highly modular NHC-N donor ligand which was used for the preparation of novel rhodium complexes for hydrosilylation reactions was reported by Ruiter et al. in 2020 [218]. This novel ligand which is based on imidazo [1,5-a]pyridine-3-ylidene, was synthesized in three steps involving Schiff base condensation of a cyano-3-pyridinecarboxyaldehyde, cyclization of the resulting diimine ligand with chloromethyl ethyl ether and subsequent reaction of cyano group with an amino alcohol which gave oxazoline functionalized imidazoylidene ligand. The structure of its trigonal bipyramidal rhodium complex is shown in Scheme 68. Tunability of the developed ligand, in particular with oxazoline moiety, allowed for the screening of a variety of rhodium complexes in the hydrosilylation of ketones. All metal complexes depicted in Scheme 68 showed high activity in the hydrosilylation of substituted ketones [218]. 

Another interesting ligand with hydroxyl functionalized NHC ligand which is used to support nickel ions to develop alternative catalysts for noble metal-based catalysts in the hydrosilylation reactions was reported in 2020 [219]. 

**Scheme 69:**
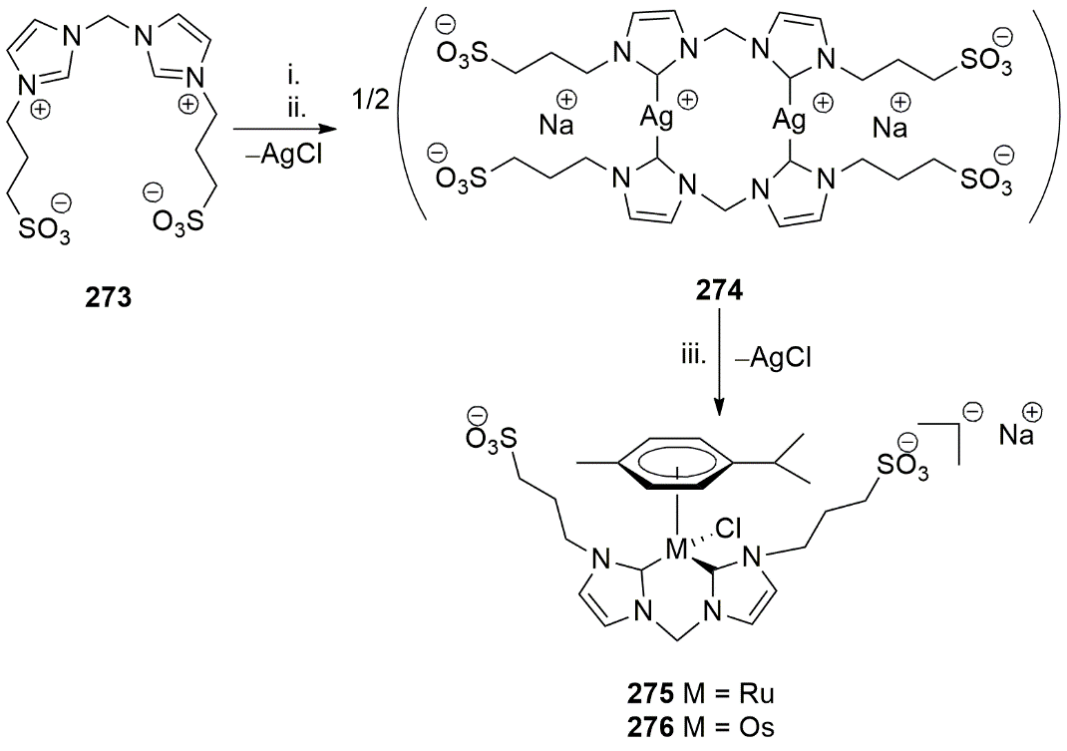
Chelation of sulfonated bis-NHC ligand [253]. Reagent and conditions: (i) 1 eq Ag_2_O; (ii) 1 eq NaCl(aq), 50 °C; (iii) 0.5 eq [(*p*-cymene)MCl_2_]_2_ (M = Ru, Os), 50 °C.

Considering green chemistry aspects of water as solvent, a variety of strategies were developed to synthesize metal complexes of NHC-based bidentate or tridentate ligands with high solubility in water. The sulfonated NHC-NHC type donor **273** for the synthesis of water soluble ruthenium(II) and osmium(II) complexes was reported by Kuhn et al. [253]. Ring opening of 1,3-propanesulfone with methylene-bridged bis(imidazolium) in boiling acetone was utilized as an efficient strategy to develop such chelating ligand [254]. As shown in Scheme 69, reaction of silver oxide with imidazolium salts **273** resulted in binuclear silver bis(NHC) complex **274**. Subsequent transmetalation with ruthenium and osmium metal salts yielded complexes **275** and **276**. 

**Scheme 70:**
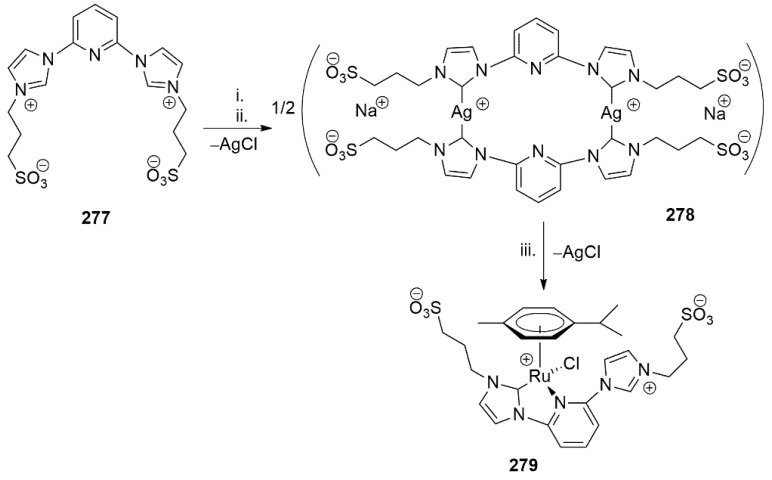
Chelation of sulfonated NHC-pyridine ligand [253]. Reagent and conditions: (i) 1 eq Ag_2_O; (ii) 1 eq NaCl(aq), 50 °C; (iii) 0.5 eq [(*p*-cymene)RuCl_2_]_2_, 50 °C.

Utilizing the same synthetic approach, the sulfonated NHC-pyridine pincer ligand **277** was synthesized to stabilize ruthenium(II) and osmium(II) ions (Scheme 70). Single crystal X-ray structure of the Ru complex **279** revealed a pseudo-tetrahedral geometry around the metal center by coordination of the NHC, the chelating pyridine, the chloride, and *p*-cymene.

Because of high solubility of these ruthenium complexes in water they were considered as potential prodrugs in medicine or in water as soluble catalysts. 

**Scheme 71:**
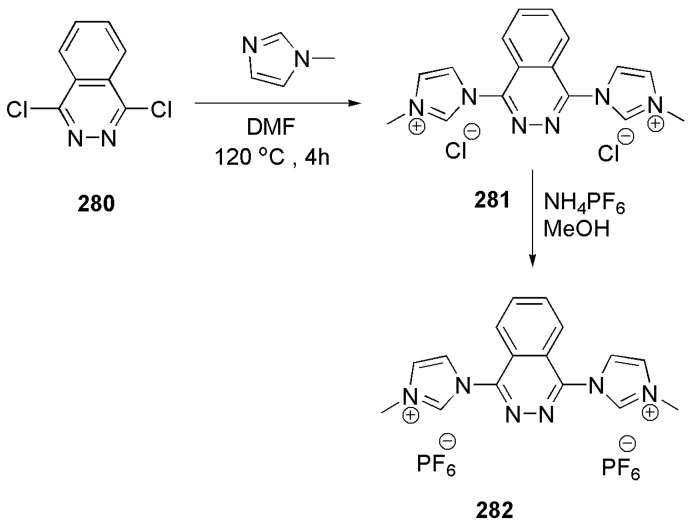
Synthesis of bis(NHC) type ligand [244].

To design a catalytic system for 2-electron oxidation processes, Sala et al. have utilized phthalazine as starting material for the synthesis of a potentially multidentate NHC ligand with chelating nitrogen donors [244]. As shown in Scheme 71, nucleophilic substitution of 2,4 dichlorophthalazine **280** by two equivalents of methyl imidazole provides a CNNC type donor ligand **281** which can easily undergo counterion exchange reaction with hexafluorophosphate to afford the stable ligand **282**. 

**Scheme 72:**
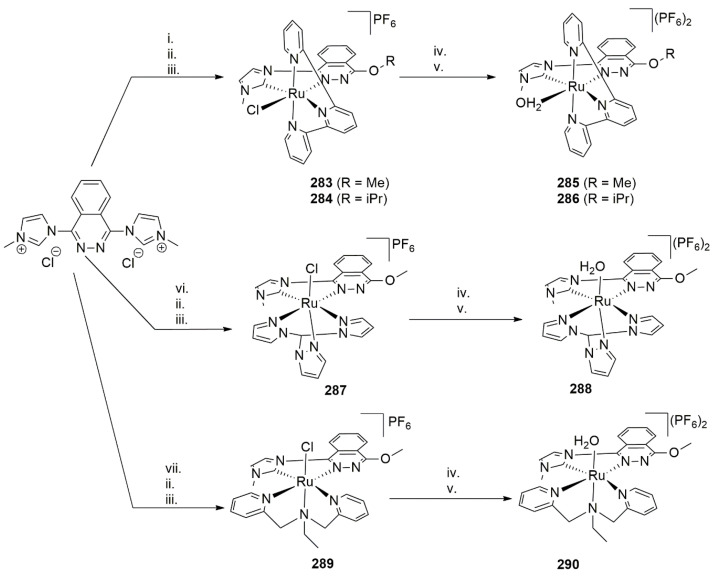
Coordination of bis(NHC) ligand to ruthenium [244]. Reagents and conditions: (i) [Ru(trpy)Cl_3_], trpy = terpyridine; (ii) Et_3_N/LiCl; (iii) NH_4_PF_6_, ROH, 80 °C, 16 h (iv) AgBF_4_; (v) NH_4_PF_6_, acetone/H_2_O, 90 °C, 4 h; (vi) [Ru(tpm)Cl_3_], tpm = tris(pyrazol-1-yl)methane; (vii) [Ru(bpea)Cl_3_], bpea = *N*,*N*-bis(pyridin-2-ylmethyl)ethanamine.

The metalation of the tetradentate ligand **282** with Ru species has led to surprising results. Reaction of 2 equivalents of **282**, regardless of the type of ruthenium precursor, yielded mononuclear ruthenium complexes in which one imidazole ring is replaced by methoxy group (from methanol solvent, Scheme 72). This reactivity represents a rare case wherein a potentially tetradentate ligand is turned into a chelating NHC-nitrogen donor ligand by virtue of solvent assisted ligand decomposition. Complexes **283**, **284**, **287**, and **289** in Scheme 72, represent octahedral mononuclear ruthenium complexes, which were further derivatized to provide aqua coordinated octahedral complexes (**285**, **286**, **288**, **290**) for water oxidation reactions.

**Scheme 73:**
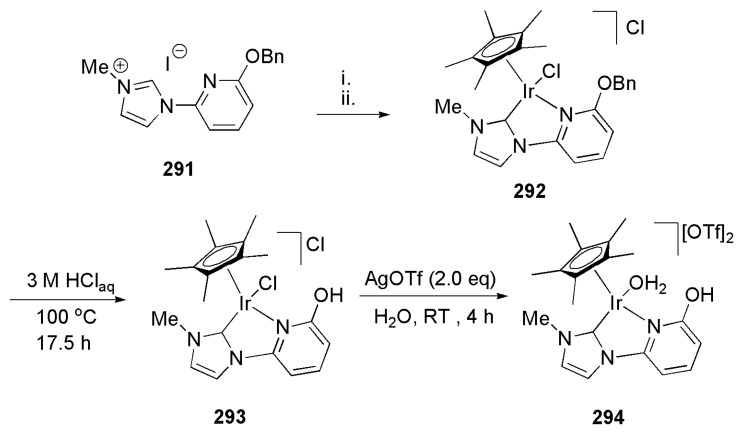
Synthesis of iridium complex supported with bidentate NHC-pyridine ligand [240]. Reagents and conditions: (i) Ag_2_O (0.50 eq), 4 h; (ii) [Cp*IrCl_2_]_2_ (0.5 eq), 4 h, CH_2_Cl_2_, r.t, Cp* = pentamethylcyclopentadiene.

As remarked above, bidentate N-heterocyclic carbene ligands have the common property of coordinating strongly to metal ions, therefore providing a metal complex with structural integrity suitable for practical applications. This characteristic was invoked in an interesting work by Yamaguchi et el. in 2017 where they have reported the synthesis of a water soluble Ir(III) metal complex bearing NHC-hydroxy pyridine bidentate ligand which is highly stable even at elevated temperatures [240]. The synthesis and metalation of this efficient catalytic system is shown in Scheme 73. The imidazolium salt **291** is transferred to an iridium metal center utilizing standard silver oxide mediated route. Subsequent benzyl group deprotection of pyridine moiety and anion exchange reaction leads to the formation of the highly stable dicationic iridium complex **294**, which is highly soluble in water. 

Complex **294** showed high catalytic activity in aqueous media for the dehydrogenative oxidation of secondary alcohols to ketones [240].

**Figure 31:**
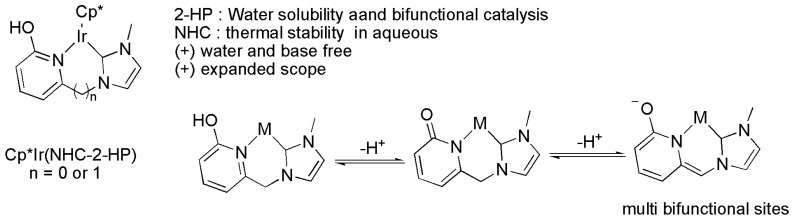
Design of bifunctional Cp*Ir complexes [245]. Cp* = Me_5_-Cp.

Ke et al. [245] reported an analogous ligand system based on 2-hydroxypyridine tethered NHC ligand. In addition, in this case, the presence of hydroxyl group on tethered pyridine was envisaged for the preparation of water-soluble metal complexes to be used in the reduction of ketones. Similar metal complexes could also act as bifunctional catalysts for N-monoalkylation of alcohols, as shown in Figure 31. Deprotonation of hydroxyl group in 2-hydroxypyridine tethered NHC ligand allows proton TH via cooperation of metal and ligand centers [245]. A pictorial description of this phenomenon is given in Figure 31.

**Scheme 74:**
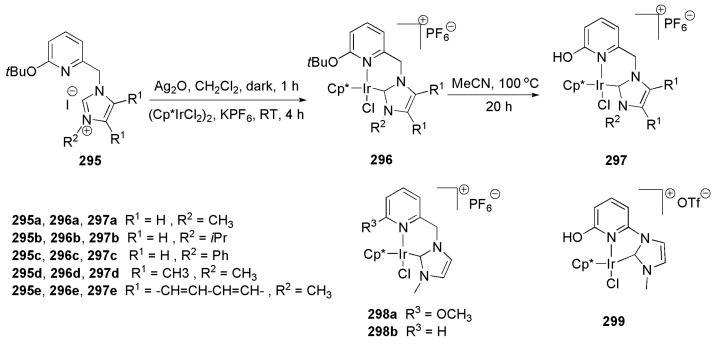
Synthesis of Ir complexes [245]. Cp* = Me_5_-Cp.

The synthesis of NHC-iridium complexes is shown in Scheme 74. Iridium complex **296** was prepared from **295** using silver oxide via transmetalation procedure, followed by the addition of [Cp*IrCl_2_]_2_. The deprotection of *t*butyl protecting group to give **297** was carried out in refluxing acetonitrile [245]. The experimental and computational studies on this particular catalytic system showed the bifunctional nature of the bidentate NHC-pyridine ligand via an outer-sphere mechanism.

**Scheme 75:**
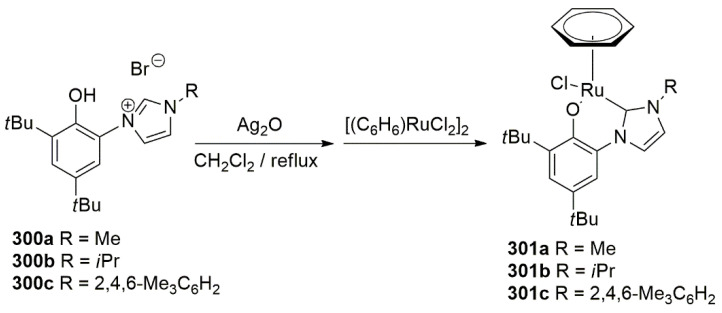
Synthesis of ruthenium complexes bearing NHC-aryloxide ligand [234].

The aryloxide-tethered NHC ligands **300a**–**c** and their ruthenium(II) complexes **301a**–**c** (Scheme 75) were described by Wang et al. [234]. In the molecular structure of **301a**, ruthenium complex adopts a piano-stool type structure in which benzene is an η^6^ bonding ligand. **301a**–**c** exhibited high activity and efficiency toward ring opening metathesis polymerization of norbornene and copolymerization of norbornene and cyclooctene [234].

**Scheme 76:**
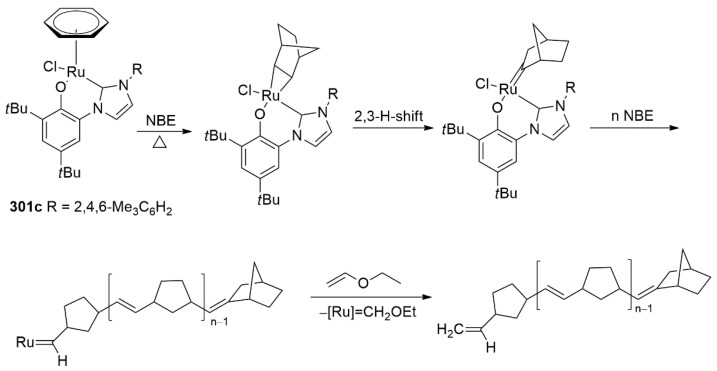
Postulated norbornene polymerization by complex **301c** [234].

The postulated mechanism for the polymerization of norbornene utilizing **301a**–**c** is shown in Scheme 76 [234]. Despite overall structural similarities, catalyst **301c** with bulky mesityl substituents showed better activities in norbornene polymerization. This observation, along with the fact that these complexes are less active than original Ru-cymene based catalysts in norbornene polymerization, revealed that an easy initial release of benzene ligand is crucial in the overall reactivity of the catalyst. The loss of benzene ligand and subsequent norbornene coordination to the metal center to initiate ring-opening polymerization reactions were shown to be a general tendency in these types of reactions [234].

**Scheme 77:**
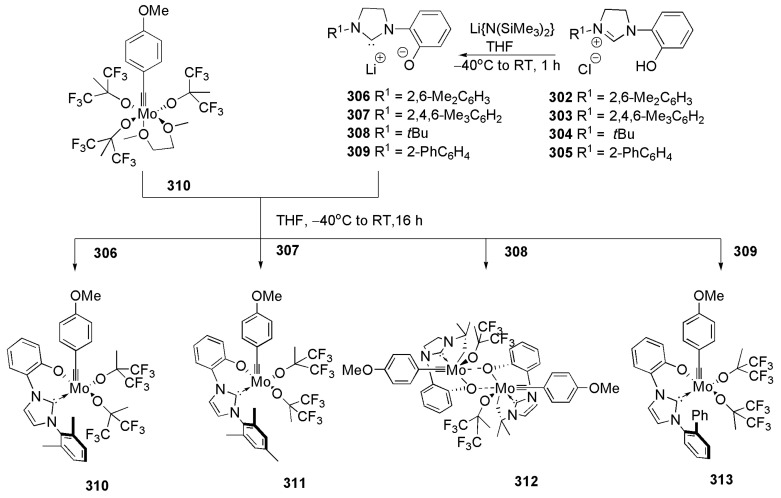
Synthesis of molybdenum alkylidyne complexes with bidentate NHCs [246].

Molybdenum and tungsten alkylidyne complexes have been largely investigated as catalysts for alkyne metathesis reactions. Due to their strong σ donating properties, bidentate N-heterocyclic carbene ligands were recently utilized to stabilize alkylidyne molybdenum and tungsten cations [255]. While there are numerous examples of metal complexes of these ligands, there are few well-defined complexes which are structurally characterized and for which the mechanistic pathways for the metathesis reactions are well understood [246]. The general structure of the complex, consists of a five coordinate 14-electron molybdenum alkylidyne complex bearing alkoxy and carbene ligands which is active in alkyne metathesis reactions. Different R (*para*-methoxy phenyl) and R^F^ (such as tris(hexafluoro-*t*butoxide) or pentafluorophenoxide have been utilized, Scheme 77. 

Mechanistic studies on these complexes have shown that either one of alkoxide or carbene ligands can dissociate and form either quasicationic or neutral active catalytic species. To clarify this hypothesis, Buchmeiser et al. prepared bi-, and tridentate molybdenum-alkylidene NHC complexes shown in Scheme 77. Four chelating NHCs were synthesized and stereoelectronic properties of the NHCs were varied by altering the nonchelating substituent from the *N*-*t*butyl (**308**) to the *N*-2,6-dimethylphenyl (**306**), the *N*-2,4,6-trimethylphenyl (**307**), and the *N*-2-phenylphenyl (**309**). Carbene donors of these imidazolium salts were synthesized prior to metal complex formation by deprotonation of the imidazolinium salts with LiN(SiMe_3_)_2_}. Depending on the noncoordinating substituent on NHC ligand, different yields from substitution of one alkoxy and dimethylethoxy ligands were obtained. In the case of *N*-tert-butyl, a C-H bond activation and subsequent elimination of alkoxide was also observed [246]. 

The derivatized molybdenum complexes were utilized in the homometathesis of 1-phenyl-1-propyne [246]. The results from tracing products of homo-metathesis reactions showed that the presence of chelating NHCs did not promote the formation of the catalytically active cationic species.

## 3. Conclusions

The continued interest in the synthesis of novel NHC ligands and investigation into their applications is evident from an increase in the number of literature reports about this class of ligands in recent years [256,257]. Among high records of publications concerning different forms of NHC ligands, a good portion is dedicated to those with tethered anionic chelates. This review article was intended to give readers a highlight of some recent advances made in the synthesis and catalytic activity of novel bidentate ligands bearing NHC donors, as well as novel features of some transition metal ions that are supported with these ligands. In terms of applications of transition metal complexes bearing bidentate NHC ligands, in asymmetric synthesis and catalytic hydrogenations of olefins, more exciting reports will appear in the coming years. While for a long time homogeneous catalytic reactions benefited from phosphine, nitrogen, or noncarbene carbon donors such as carbonyl ligands, the structural and electronic tunabiltiy of this fascinating class of ligands allowed their powerful entrance to the filed of catalytic homogeneous reactions. Despite this, inherent differences between NHCs with phosphines or carbonyls such as lack of orthogonal reactivity, due to inherent planar nature of NHC ligands, or lack of strong π acidity in the majority of reported cases so far, delayed their utility in some catalytic reactions. Solution to these issues would boost applications of these fascinating class of ligands even further.

## Data Availability

Not applicable.
